# The Synthesis of Poly(Vinyl Alcohol) Grafted with Fluorinated Protic Ionic Liquids Containing Sulfo Functional Groups

**DOI:** 10.3390/molecules26144158

**Published:** 2021-07-08

**Authors:** Patrycja Glińska, Andrzej Wolan, Wojciech Kujawski, Edyta Rynkowska, Joanna Kujawa

**Affiliations:** 1 Faculty of Chemistry, Nicolaus Copernicus University in Toruń, 7 Gagarina Street, 87-100 Toruń, Poland; patrycjaglinska1996@gmail.com (P.G.); wolan@chem.umk.pl (A.W.); edyta.rynkowska@wp.pl (E.R.); joanna.kujawa@umk.pl (J.K.); 2 Synthex Technologies Sp. z o.o., 7 Gagarina Street, 87-100 Toruń, Poland

**Keywords:** poly(vinyl alcohol), chemical modification, protic ionic liquid, imidazolium-based ionic liquid

## Abstract

There has been an ongoing need to develop polymer materials with increased performance as proton exchange membranes (PEMs) for middle- and high-temperature fuel cells. Poly(vinyl alcohol) (PVA) is a highly hydrophilic and chemically stable polymer bearing hydroxyl groups, which can be further altered. Protic ionic liquids (proticILs) have been found to be an effective modifying polymer agent used as a proton carrier providing PEMs’ desirable proton conductivity at high temperatures and under anhydrous conditions. In this study, the novel synthesis route of PVA grafted with fluorinated protic ionic liquids bearing sulfo groups (–SO_3_H) was elaborated. The polymer functionalization with fluorinated proticILs was achieved by the following approaches: (i) the PVA acylation and subsequent reaction with fluorinated sultones and (ii) free-radical polymerization reaction of vinyl acetate derivatives modified with 1-methylimidazole and sultones. These modifications resulted in the PVA being chemically modified with ionic liquids of protic character. The successfully grafted PVA has been characterized using ^1^H, ^19^F, and ^13^C-NMR and FTIR-ATR. The presented synthesis route is a novel approach to PVA functionalization with imidazole-based fluorinated ionic liquids with sulfo groups.

## 1. Introduction

Proton exchange membrane (PEM) is the key component of the fuel cell, being responsible for the transport of protons through the polymeric membrane from anode to the cathode, which enables efficient operation of proton exchange membrane fuel cell (PEMFC). The desired properties of PEM combine high proton conductivity, chemical and thermal stability in fuel cell operating conditions, mechanical strength, and long lifespan [1,2]. Many attempts have been studied in order to maximize efficiency and to reduce the cost of PEMs by physical and chemical modification of polymers such as polyethylene (PE) [3], polybenzimidazole (PBI) [4], poly(arylene ether sulfone) (PAES) [5], and poly(vinyl alcohol) (PVA) [6] using different additives such as nanoparticles and protic ionic liquids (proticILs) [7,8].

Over the years, researchers have been developing the functionalization of polymer materials by applying various approaches, including synthesis, grafting, crosslinking, and polymerization [3,7,9,10,11,12,13,14,15]. Such modification methods result in the formation of chemical bonds between the polymer and the modifying agent. Elumalai et al. [11] proposed the preparation method of poly(styrene-ethylene/butylene-styrene) block copolymer (PSEBS) functionalization using phosphonic acid via a two-step approach: PSEBS chloromethylation followed by its phosphonation utilizing the Michaels–Arbuzov reaction. The researchers found that the functionalized PSEBS-based membranes possessed a high tensile strength equal to 2.9 MPa, thermal stability up to 400 °C, and proton conductivity of 5.81 mS cm^−1^ at 140 °C [11]. Kim et al. [3] elaborated on copolymer of polyethylene (PE) and sulfonated poly(arylene ether sulfone) (PAES) via graft-onto reaction (PE-g-PAES). The synthesis route involved the reaction between PE molecules and pendent benzyl bromide groups PAES with two terminal phenol groups. The use of two polymers provided the combination of hydrophobicity, semicrystallinity, and high molecular weight, which resulted in the non-swellable PE-g-PAES-based PEM of proton conductivity up to 160 mS cm^−1^ at 120 °C [3]. Zeng et al. [10] developed the method of PAES functionalization with perfluoroalkyl sulfonic acids (PSA) via polymer post modification using Ullmann coupling reaction with potassium 1,1,2,2-tetrafluoro-2-(1,1,2,2-tetrafluoro-2-iodoethoxy)ethanesulfonate (PSA-K). PAES-PSA membranes modification improved proton conductivity up to 12.69 × 10^−2^ S cm^−1^ compared to Nafion 117 (9.55 × 10^−2^ S cm^−1^) at 60 °C [10]. Bai et al. [7] studied high-temperature PEM polysulfone-based membrane grafted with poly(1-vinylimidazole) via atom transfer radical polymerization and subsequently activated with phosphoric acid. The elaborated PEM revealed the high proton conductivity equal to 127 mS cm^−1^ at 160 °C and tensile strength equal to 7.94 MPa [7].

The special attention of researchers towards functionalized polymer PEMs has been addressed to materials based on PVA or derivatives thereof [16]. Poly(vinyl alcohol) has been investigated as a non-perfluorinated polymer for PEM due to its low price, biodegradability, harmless nature, high thermal stability and chemical resistance, high flexibility, and good film-forming properties [6,15]. PVA is a semi-crystalline synthetic polymer characterized by numerous properties, such as high hydrophilicity, water solubility, good film/gel-forming, adhesive and emulsifying properties [12,17,18,19]. PVA is also a non-toxic, biocompatible, and biodegradable polymer with high barrier properties towards oxygen that make it suitable for many industrial applications [17,20,21,22]. Moreover, PVA is characterized by the high dielectric permittivity and charge storage capacity, which make it a suitable polymer for application as a supercapacitor [6]. However, ion carriers such as sulfosuccinic acid (SSA) [9] or protic ionic liquids (proticILs) [6] must be introduced to PVA in order to prepare PEM.

Albayrak Arı et al. [13] proposed the synthesis of imidazolium-functionalized PVA by acetalization and direct quaternarization reactions. The obtained functionalized PVA containing the imidazolium and quaternary ammonium functional groups was subsequently applied on the porous polycarbonate (PC) membranes for direct methanol fuel cell application. The researchers reported the superior performance efficiency of imidazolium over ammonium-based composites. A PVA/PC membrane with imidazolium functional groups possesses high ionic conductivity (7.8 mS cm^−1^ at 30 °C). Boroglu et al. [23] elaborated PVA-based PEMs sulfonated with SSA (sulfosuccinic acid) to ensure a proton source and crosslinked membrane matrix and filled with imidazole to enable the proton transfer in the anhydrous conditions. The obtained PVA-SSA-Im materials for direct methanol fuel cell application were thermally stable up to 200 °C and possessed the proton conductivity equal to 1.4 mS cm^−1^ at 140 °C. 

An interesting approach has been also presented by Jansen and co-workers [24], who developed fluoropolymer gel membranes based on pure and mixed ionic liquids. As a fluoropolymer substrate, the poly(vinylidene fluoride-co-hexafluoropropylene) has been selected. Authors investigated two various ionic liquids: 1-ethyl-3-methylimidazolium bis(trifluoromethylsulfonyl)imide and 1-hexadecyl-3-methylimidazolium bis(trifluoromethylsulfonyl)imide. The ionic liquids fillers were applied either in a pure form or with a mixture of both. Independently from the selected form of ionic liquids, the addition to the polymer ranged between 20 and 80 wt%. The selection of the fillers has been done based on their chemical nature, e.g., presence of fluorinated bis(trifluoromethylsulfonyl)imide, ensuring better affinity between the ionic liquids and highly fluorinated polymer. The presented approach enhanced the membrane performance assessed during the pervaporation process of limonene/carvone mixtures separation. Due to the improved features of the separation materials, the pure gas permeability and selectivity were been boosted. The supported ionic liquid membranes applying ceramic nanofiltration membranes and tetrapropylammonium tetracyanoborate were also tested for removal of 1,3-propanediol from an aqueous solution, which is significant in the biotransformation processes fermentation broth from K. pneumoniae [25]. The authors pointed out an increase of separation factor from 0.4 to 177 with a simultaneous substantial flux decline (from 34.3 to 3.86 g m^−2^ h^−1^).

ProticILs are compounds with proven usefulness in organic chemistry and material engineering. They are excellent catalyst carriers, electrolytes, and polymeric membranes components for a wide range of applications. Therefore, ProticILs have a great potential in proton exchange membranes elaboration for fuel cells application [26,27]. Fluorinated ionic liquids have attract special attention owing to the high oxidative stability enabling the suppression of oxide formation on Pt and improved cathode performance [28]. Thomson et al. [28] reported the 2-fluoropyridinium triflate (2-FPTf), proton-conducting ionic liquid, as a more efficient electrolyte compared to the commonly used aqueous concentrated (85%) phosphoric acid. However, the main drawback of the polymeric membrane materials possessing the ionic liquids is the gradual and uncontrollable leaching out of ionic liquid from the membrane structure during the exploitation [29]. This fact constitutes a major problem leading to an efficiency and performance deterioration of the fuel cell using polymer-ionic liquid membrane electrolyte.

Recognizing the diverse characteristics of ionic liquids and perfluorosulfonated acid-based compounds and the importance of the chemical polymer modification, this work aimed to minimize the problem of progressive and uncontrollable loss of the ionic liquid from a polymer matrix. The poly(vinyl alcohol)/PVA/grafted with fluorinated protic ionic liquids functionalized with sulfo groups was designed and elaborated, synergizing the attractive properties of fluorinated ionic liquids and PVA. The objective was to obtain the PVA-proticIL systems for the potential application as PEM where a proticIL is bounded to the polymer matrix by the polymer chemical modification. PVA modification was achieved by (i) PVA acylation and subsequent reaction with fluorinated sultones and (ii) free-radical polymerization reaction of vinyl acetate derivatives modified with 1-methylimidazole and sultones. PVA functionalization was confirmed using ^1^H and ^19^F-NMR, as well as FTIR-ATR analysis.

## 2. Results and Discussion

In the first stage, the method of PVA acylation with a subsequent reaction with fluorinated sultones was developed. Due to the limited solubility of PVA in solvents other than water, this modification was challenging to carry out and required a mixture of *N*,*N*-dimethylacetamide and anhydrous lithium chloride. Using a system consisting of *N*,*N*-dimethylacetamide and lithium chloride allowed the dissolution of PVA at a relatively low temperature and a short reaction time, which prevented hydrolysis and subsequent reactions of active halogen atoms in the alpha position to the carbonyl group. The solvent’s polar nature decreased the electron density on the carbonyl carbon atom in the presence of the lithium chloride salt, which tended the formation of a transition state with hydroxyl oxygen atom of PVA, giving a residual positive charge. This attracted σ-electrons in the polymer chain by an inductive effect, thereby increasing the electron density near the hydroxyl group, hence causing shielding, which confirmed the above transition state of PVA in the said solvent system.

**Scheme 1:**
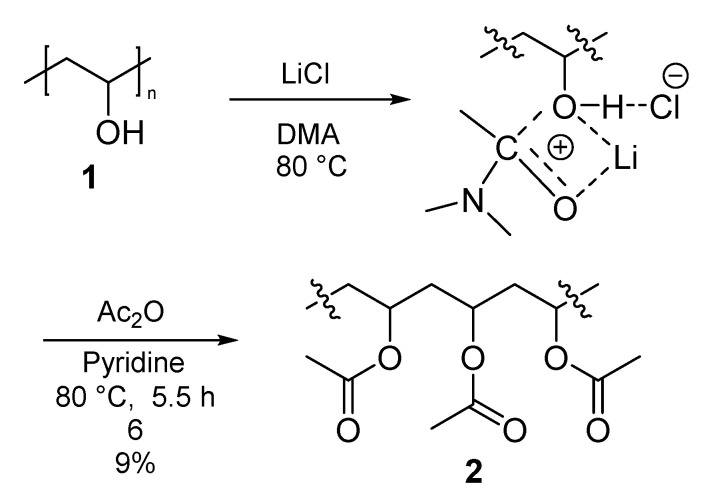
Acylation of PVA with acetic anhydride.

At 80 °C, PVA dissolves in this mixture and undergoes the acylation with acetic anhydride in good yield (Scheme 1). The structure of polymer I (**2**) can only be confirmed by utilizing the IR spectrum (Figure S1) because of the insolubility of the product in the following six common solvents used for NMR spectra: DMSO, chloroform, methanol, toluene, acetone, and THF. The presence of a strong band at 1731 cm^−1^ confirms the presence of a C=O bond from the ester group.

**Scheme 2:**
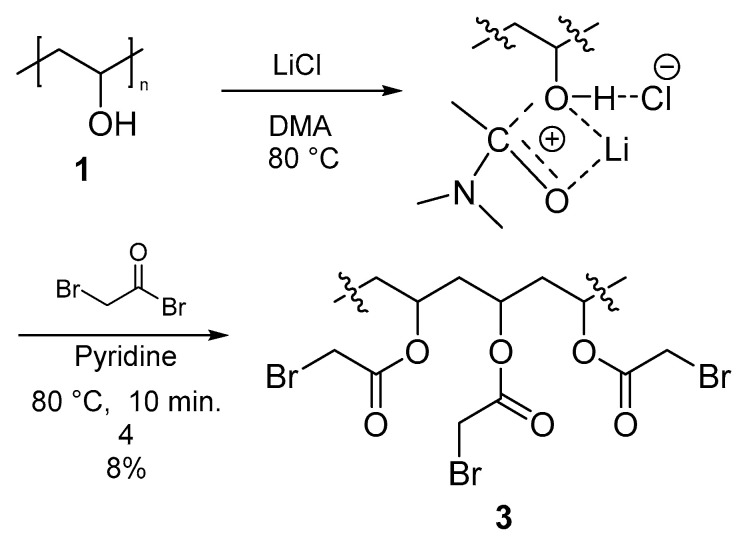
Acylation of PVA with bromoacetyl bromide.

Subsequently, the previously developed acylation conditions were used in the reaction with bromoacetyl bromide (Scheme 2). The intended polymer II (**3**) was obtained, as evidenced by the IR spectrum (Figure S2) and the band at 1731 cm^−1^, indicating the presence of the ester group. However, the process yield was low, and the resulting product mixture was contaminated.

**Scheme 3:**
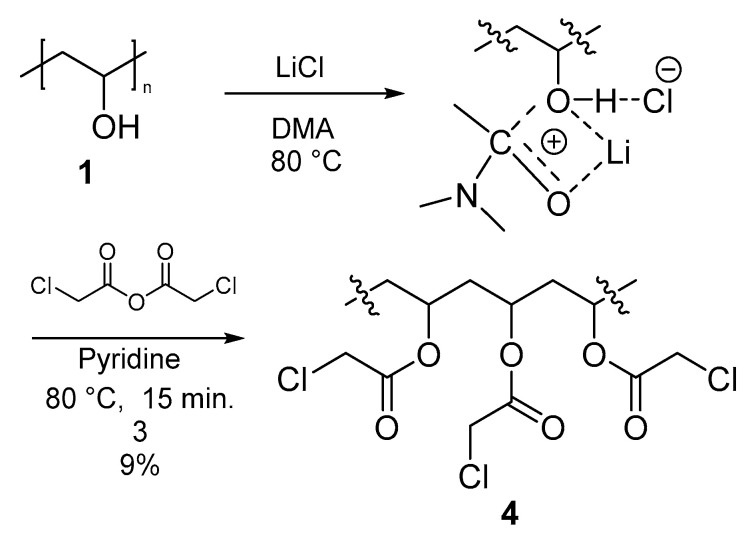
Acylation of PVA with 2-chloroacetic anhydride.

As a result, 2-chloroacetic anhydride was used as another acylating agent. The use of 2-chloroacetic anhydride resulted in a less rapid reaction and lower yield; however, polymer III—product (**4**) was obtained with higher purity (Scheme 3, Figure S3).

**Scheme 4:**
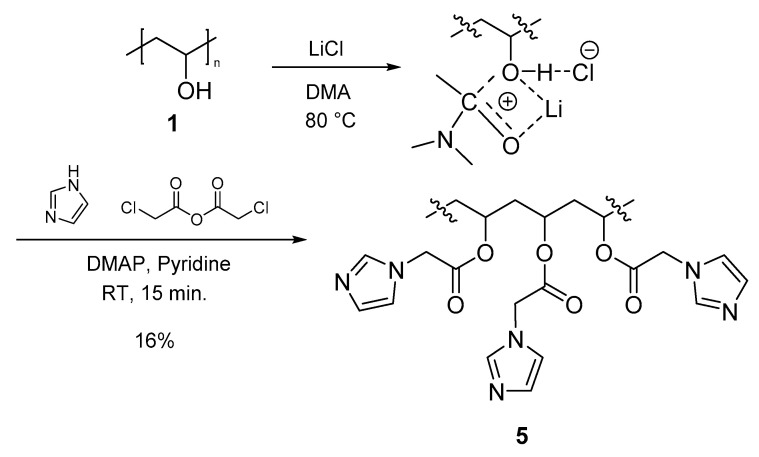
Acylation of PVA with 2-chloroacetic anhydride in the presence of imidazole.

The product of the PVA acylation was foreseen to be a substrate in the reaction with imidazole. Due to the insolubility of acylated PVA in any common solvent that could be used in the reaction with the imidazole sodium salt (DMSO, chloroform, methanol, toluene, aceton, and THF), it was decided to perform the PVA reaction with 2-chloroacetic anhydride in the presence of imidazole. As a result, the polymer IV (**5**) shown in Scheme 4 was obtained. The IR spectrum of (**5**) is presented in Figure S4.

The degree of substitution that can be obtained using acetic anhydride and acetyl chloride is 0.94 and 0.96, respectively, which means that virtually every hydroxyl group is substituted, as shown by Tosh et al. [30]. Since the PVA derivatives obtained in this work were insoluble, it was challenging to determine the degree of substitution by NMR. Therefore, the degree of substitution was determined by elemental analysis, measuring the bromine and chlorine content, respectively. A practically complete substitution was achieved, expressed as a degree of substitution of 96% and 98% for the bromo- and the chloro-derivative, respectively, using an excess of the acylating agent. The obtained results are consistent with the work of Jantas et al. [31]. Using a similar method, the researchers achieved a 98.6% degree of substitution of OH groups in PVA using 1.2 equivalents of chloroacetic acid chloride [31].

**Scheme 5:**
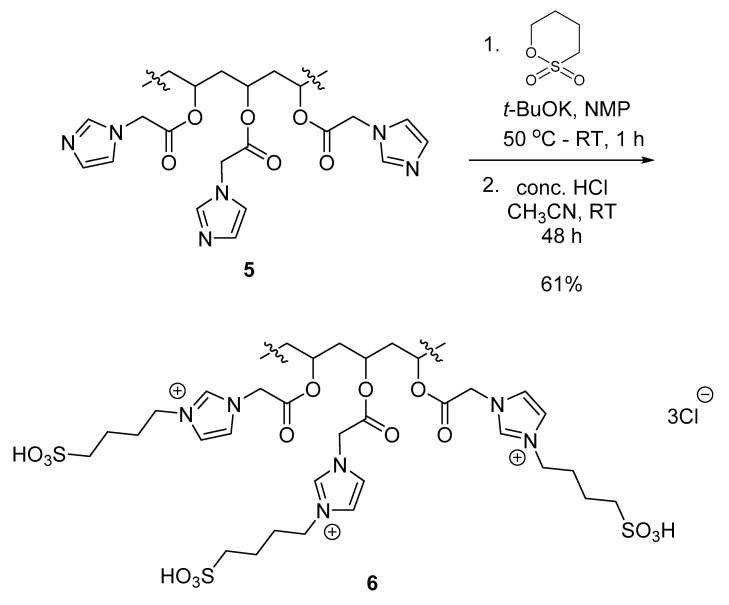
Reaction of acylated PVA with 1,4-butanesultone.

The next step of the PVA functionalization was the reaction of the above-modified PVA with 1,3-propane sultone and 1,4-butane sultone. The reaction was carried out repeatedly, using various solvents, as well as without solvents, in an excess of sultone. Due to the low solubility of reagents in all six tested solvents, the intended polymer V—product (**6**) was obtained with a moderate overall yield (Scheme 5, Figure S5).

In the second stage, the free-radical polymerization reaction of vinyl acetate derivatives modified with 1-methylimidazole and sultones was elaborated. It was decided to modify the corresponding vinyl acetate derivatives and then to carry out the polymerization reaction to obtain functionalized PVA due to the limited solubility of PVA and its derivatives in organic solvents.

**Scheme 6:**
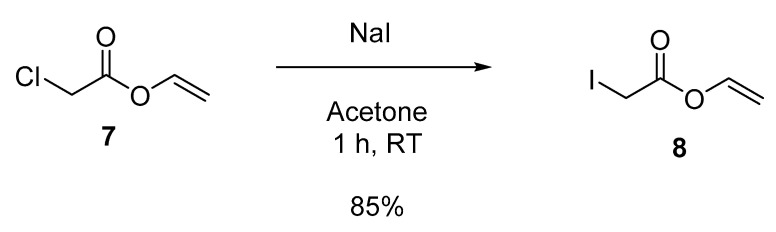
Synthesis of vinyl iodoacetate (**8**).

First, vinyl iodoacetate (**8**) was obtained with 85% yield by reacting vinyl chloroacetate with sodium iodide in acetone (Scheme 6, Figure S6).

**Scheme 7:**
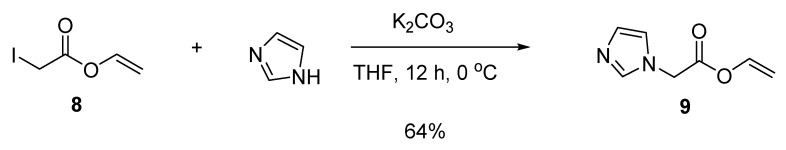
Synthesis of vinyl 2-(1H-imidazol-1-yl)acetate (**9**).

Subsequently, the reaction between vinyl iodoacetate (**8**) and imidazole in the presence of potassium carbonate in tetrahydrofuran solution was carried out with 64% yield, resulting in the synthesis of vinyl 2-(1H-imidazol-1-yl)acetate (**9**) (Scheme 7, Figure S7).

**Scheme 8:**
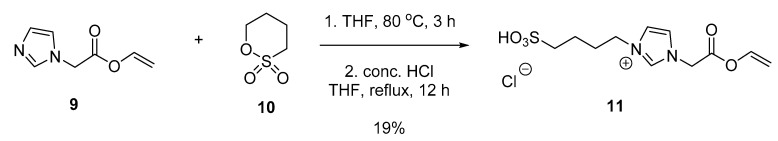
Synthesis of sulfonated imidazolium-based ionic liquid (**11**) in the reaction of vinyl 2-(1H-imidazol-1-yl)acetate and 1,4-butanosultone.

**Scheme 9:**
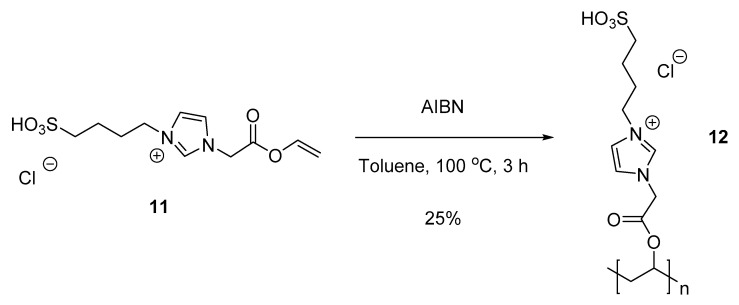
Synthesis of polymer V (**12**).

In the next step, reaction with 1,4-butanosultone was performed to obtain an ionic liquid (**11**) according to Scheme 8 (Figure S8):

The free radical polymerization of the obtained sulfonated ionic liquid was limited due to its poor solubility in toluene. Nonetheless, the product (**12**) presented in Scheme 9 (Figure S9) was obtained.

**Scheme 10:**
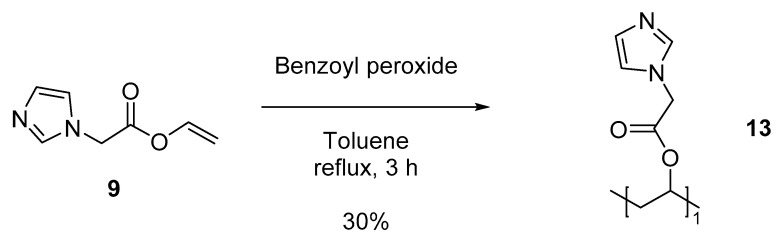
Polymerization of vinyl 2-(1H-imidazol-1-yl)acetate.

**Scheme 11:**
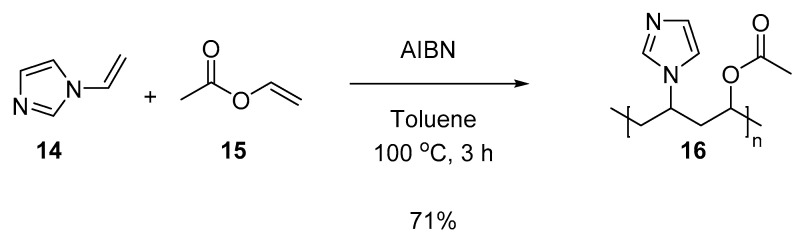
Copolymerization of *N*-vinyl imidazole and vinyl acetate.

The product of the above reaction was hardly soluble in all six tested solvents; therefore, the membrane preparation by a phase inversion method may be problematic. Consequently, it was decided to obtain two more polymers, i.e., (**13**)—Figure S10—and (**16**)—Figure S11—according to the following approaches: by a polymerization of vinyl 2-(1H-imidazol-1-yl)acetate (Scheme 10) and by a copolymerization of *N*-vinyl imidazole and vinyl acetate (Scheme 11).

**Scheme 12:**
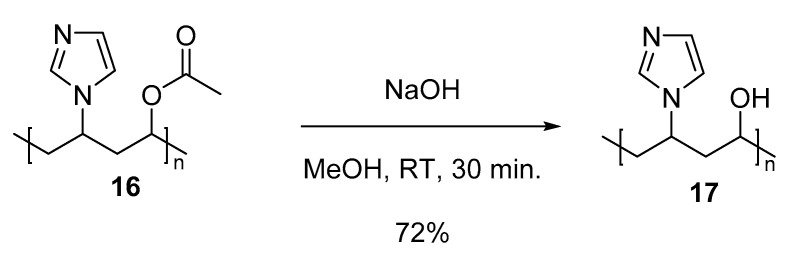
Hydrolysis of acetyl group in copolymer I (**16**).

Hydrolysis of the acetyl group under basic conditions resulted in a copolymer II (**17**) containing an active hydroxyl group and an imidazole ring (Scheme 11, Figure S12), which can be nucleophiled by reaction with the appropriate sulfones (Scheme 12). The ratio between the imidazole ring and active hydroxyl groups was determined by elemental analysis and is equal to approximately 1:1.

**Scheme 13:**
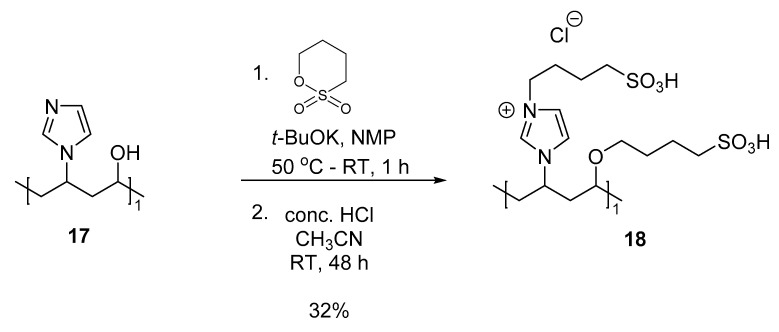
Reaction of copolymer II (**17**) with 1,4-butane sultone.

The copolymer II (**17**) reacts with 1,4-butane sultone, resulting in a copolymer III (**18**) modified with a protic ionic liquid after an anion exchange step (Scheme 13, Figure S13).

**Scheme 14:**
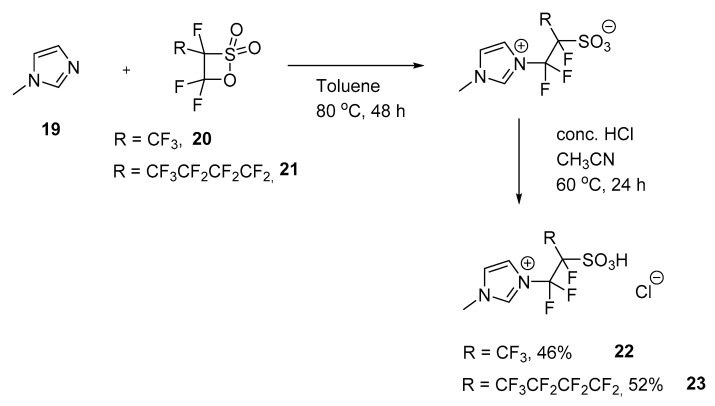
Synthesis of 1-methyl-3-(perfluoro-2-sulfoethyl)-1H-imidazol-3-ium chlorides.

Ethanosultones underwent the reaction with N-methylimidazole, followed by anion exchange with the hydrochloric acid, to test the possibility of using fluorinated sultones to modify the polymer (Scheme 14, Figures S14–S17). The products (**22**) and (**23**) were obtained with moderate yields (46% and 52%, respectively); nevertheless, this reaction confirmed the possibility of using such perfluorinated sultons for the quaternization of the imidazole ring.

**Scheme 15:**
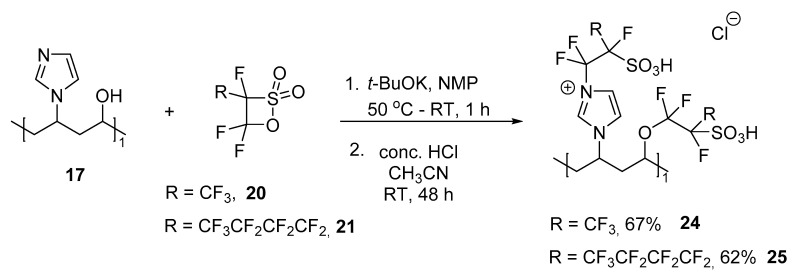
Reaction of copolymer II with perfluorinated sultones.

The final step was the use of perfluorinated etanesultones to functionalize copolymer II (**17**)—Scheme 15. As a result of this reaction, copolymers (**24**) and (**25**) bearing a sulfonic acid group on both the hydroxyl group and the imidazole ring were obtained (Figures S18 and S19). The products (**24**) and (**25**) were synthesized with good yields of 67%. This synthesis yield, however, was lower compared to the yield of reaction with 1,4-butane sultone, due to the competitive elimination response promoted by the base.

The main problem that may arise in a nucleophile reaction with perfluorinated ethylene sultone derivatives is the elimination reaction of hydrogen fluoride and rearrangement to the corresponding carbonyl compounds. Nevertheless, when using nucleophiles that are not strong bases, this problem can be avoided, and appropriate sultone ring-opening products can be obtained, as exemplified by the publications in which the authors obtain suitable ethers maintaining the SO_3_H group and the perfluorinated alkyl chain [32,33].

## 3. Materials and Methods

### 3.1. Materials

Experiments with air and moisture-sensitive materials were carried under argon atmosphere. Glassware was oven-dried for several hours, assembled hot, and cooled in a stream of argon. Silicagel 60, Merck 230–400 mesh, was used for the preparative column flash chromatography. Analytical TLC was performed using Sigma Aldrich silica gel on TLC Al foils with fluorescent indicator. Lithium chloride, PVA (Elvanol^®^ 71-30), *N*,*N*-dimethylacetamide, acetic anhydride, pyridine, 2-bromoacetyl bromide, 2-chloroacetic anhydride, imidazole, *N*-methylpyrrolidone, 1,4-butanesultone, potassium t-butoxide, acetone, 1,3-propanesultone, sodium iodide, vinyl 1-chloroacetate, potassium carbonate, tetrahydrofuran, 2,2′-azodi(isobutyronitrile), *N*-vinylimidazole, vinyl acetate, 1,2,2-trifluoro-2-hydroxy-1-trifluoromethylethanesulfonic acid sultone, 4-dimethylaminopyridine were commercial products.

### 3.2. Characterization Methods

^1^H and ^19^F-NMR spectra were recorded on a Bruker Advance III 400 MHz or Bruker Advance III 700 MHz instrument at ambient temperature. Chemical shifts are reported in parts per million (δ scale), and coupling constants (*J* values) are listed in Hertz. IR spectra were recorded on a Bruker ALPHA spectrometer equipped in platinum Diamond ATR in the range of 4000–500 cm^−1^ with a resolution of 4 cm^−1^ and 16 scans.

### 3.3. Process of PVA Acetylation

#### 3.3.1. Polymer I (PVA-Ac) (**2**)

Acetylation of PVA with acetyl chloride (in DMA in the presence of LiCl)

Lithium chloride (0.30 g, 6.98 mmol) and PVA (**1**) (0.31 g, 6.97 mmol) were placed in a 50 mL round-bottomed flask equipped with a reflux condenser. Subsequently, *N*,*N*-dimethylacetamide (30 mL, 0.32 mol) was added. The bath temperature was set to 80 °C. The mixture was stirred at this temperature for 2 h until the PVA was completely dissolved. Next, pyridine (0.54 mL, 6.9 mmol) was added, and then acetic anhydride (0.66 mL, 6.9 mmol) was slowly added dropwise. Acetylation was carried out at 80 °C for 5.5 h. After this time, the reaction mixture was poured into 100 g of finely crushed ice. The precipitated polymer was washed with water and dried in vacuo to give 0.71 g (69%) of pale-yellow powder.

IR (ATR, cm^−1^); 3010 (m), 2921 (m), 1731 (s, C=O stretching), 1407 (s), 1310 (s), 1286 (s), 1186 (s), 1019 (m)—Figure S1.

#### 3.3.2. Polymer II (PVA-AcBr) (**3**)

Acetylation of PVA with 2-bromoacetyl bromide (in DMA in the presence of LiCl)

Lithium chloride (0.25 g, 5.80 mmol) and PVA (**1**) (0.51 g, 11.00 mmol) were placed in a 50 mL round-bottomed flask equipped with a reflux condenser. Subsequently, *N*,*N*-dimethylacetamide (25 mL, 0.26 mol) was added. The bath temperature was set to 80 °C. The mixture was stirred at this temperature for 2 h until the PVA was completely dissolved. Next, pyridine (0.85 mL, 11.00 mmol) was added and then 2-bromoacetyl bromide (0.95 mL, 11.00 mmol) was slowly added dropwise. Acetylation was carried out at 80 °C for 10 min. After this time, the reaction mixture was poured into 100 g of finely crushed ice. The precipitated polymer was washed with water and dried in vacuo to give 1.30 g (48%) of brownish powder.

IR (ATR, cm^−1^); 3331 (m), 2951 (m), 1731 (s, C=O stretching), 1635 (m), 1406 (m), 1304 (m), 1188 (s), 674 (m, C–Br stretching)—Figure S2.

Anal. Calcd for C_4_H_5_BrO_2_: C, 29.12; H, 3.05; Br, 48.43. Found: C, 29.70; H, 3.14; Br, 46.49.

#### 3.3.3. Polymer III (PVA-AcCl) (**4**)

Acetylation of PVA with 2-chloroacetic anhydride (in DMA in the presence of LiCl)

Lithium chloride (0.36 g, 7.96 mmol) and PVA (**1**) (1.16 g, 25.20 mmol) were placed in a 100 mL round-bottomed flask equipped with a reflux condenser. Subsequently, *N*,*N*-dimethylacetamide (55 mL, 0.57 mol) was added. The bath temperature was set to 80 °C. The mixture was stirred at this temperature for 2 h until the PVA was completely dissolved. Next, pyridine (2.20 mL, 25.20 mmol) was added and then 2-chloroacetic anhydride (0.95 mL, 11.00 mmol) was slowly added dropwise. Acetylation was carried out at 80 °C for 15 min. After this time, the reaction mixture was poured into 100 g of finely crushed ice. The precipitated polymer was washed with water and dried in vacuo to give 2.17 g (39%) of pale-orange powder.

IR (ATR, cm^−1^); 3347 (m), 2953 (m), 1733 (s, C=O stretching), 1612 (m), 1407 (m), 1287 (s), 1172 (s), 779 (s, C–Cl stretching)—Figure S4.

Anal. Calcd for C_4_H_5_ClO_2_: C, 39.86; H, 4.18; Cl, 29.41. Found: C, 40.45; H, 4.26; Cl, 28.82.

#### 3.3.4. Polymer IV (PVA-AcIm) (**5**)

Acetylation of PVA with 2-chloroacetic anhydride in the presence of imidazole (in DMA in the presence of LiCl)

Lithium chloride (0.16 g, 3.80 mmol) and PVA (**1**) (0.33 g, 7.4 mmol) were placed in a 100 mL round-bottomed flask equipped with a reflux condenser. Then, *N*,*N*-dimethylacetamide (16 mL, 0.16 mol) was added. The bath temperature was set to 80 °C. The mixture was stirred at this temperature for 2 h until the PVA was completely dissolved. Then, 4-dimethylaminopyridine (0.90 g, 7.4 mmol) was added and 2-chloroacetic anhydride (2.26 g, 7.4 mmol) was slowly added dropwise. Acetylation was carried out at 80 °C for 15 min, and then imidazole (0.50 g, 7.4 mmol) was added. The stirring at 80 °C was continued for 1 h. After this time, the reaction mixture was poured into 100 g of finely crushed ice. The precipitated polymer was washed with water and dried in vacuo to give 0.48 g (16%) of brownish powder.

IR (ATR, cm^−1^); 3409 (m), 2936 (m), 1744 (m, C=O stretching), 1655 (s), 1402 (s), 1300 (s), 1263 (s), 1041 (s)—Figure S4.

### 3.4. Modification of Polymer IV (PVA-AcIm) with Sultones

#### Polymer V (PVA-AcIm-1,4) (**6**)

Reaction of polymer IV (PVA-AcIm) with 1,4-butanosultone

Polymer IV (**5**) (5.90 g, 134 mmol) and *N*-methylpyrrolidone (134 mL) were placed in a 250 mL two-necked flask. The dissolution of polymer IV was carried out at 120 °C under a nitrogen atmosphere. After one hour, the temperature was lowered to 50 °C and a solution of 1,4-butanesultone (1.82 g, 13.4 mmol) in *N*-methyl-2-pyrrolidone (NMP) (24 mL) was added. Then, a solution of potassium *t*-butoxide (1.50 g, 13.4 mmol) in NMP (24 mL) was slowly added dropwise. The reaction was carried out for 1 h at room temperature. A polymer precipitated in the flask, which was filtered off and dried in vacuo. Then, the product was suspended in acetonitrile (200 mL) and concentrated hydrochloric acid was added (8.4 mL, 100 mmol). The mixture was stirred for 48 h at room temperature under nitrogen atmosphere. The precipitate was filtered off. Moreover, washed with acetonitrile, dired in vacuo to give 4.68 g (61%) of pale-yellow powder.

IR (ATR, cm^−1^); 3294 (w), 2929 (m), 1657 (s, C=O stretching), 1441 (s), 1404 (s), 1301 (s, S=O stretching), 1261 (m), 1144 (m)—Figure S5.

### 3.5. Synthesis of Vinylimidazole and Vinyl Acetate Copolymers

#### 3.5.1. Vinyl 2-iodoacetate (**8**)

Sodium iodide (3.75 g, 25 mmol) and acetone (15 mL) were placed in a 50 mL round-bottomed flask. Vinyl 1-chloroacetate (**7**) (3.00 g, 25 mmol) was slowly added dropwise. The reaction was carried out at room temperature for an hour. Then, the precipitate was filtered off and the solvent was evaporated on a rotary evaporator. The crude product was dried in vacuo to give 3.63 g (85%) of pale-yellow oil.

IR (ATR, cm^−1^); 3127 (w), 2954 (w), 1738 (s, C=O stretching), 1654 (s), 1230 (s), 1127 (s), 1080 (s), 635 (s, C–I stretching)—Figure S6.

^1^H-NMR (700 MHz, CDCl_3_), δ (ppm); 3.76 (s, 2H, CH_2_); 4.66 (dd, *J* = 6.2 Hz; 1.8 Hz, 1H, =CH_2_); 4.97 (dd, *J* = 13.8 Hz; 1.8 Hz, 1H, =CH_2_); 7.23 (dd, *J* = 13.8 Hz; 6.2 Hz, 1H, =CH).

#### 3.5.2. Vinyl 2-(1H-imidazol-1-yl)acetate (**9**)

Vinyl 2-iodoacetate (**8**) (7.31 g, 35 mmol), potassium carbonate (4.79 g, 35 mmol) and THF (20 mL) were placed in a 100 mL round-bottomed flask. The mixture was cooled to 0 °C. Then, a solution of imidazole (2.36 g, 35 mmol) in THF (20 mL) was slowly added dropwise. The reaction was carried out for 12 h at 0 °C. After this time, the precipitate was filtered off, the solvent was evaporated on a rotary evaporator and the product was dried in vacuo to give 2.63 g (64%) of pale-yellow oil.

IR (ATR, cm^−1^); 3111 (m), 2984 (m), 1759 (s C=O stretching), 1644 (s), 1420 (s), 1172 (s), 1076 (s), 1032 (s)—Figure S7.

^1^H-NMR (700 MHz, CDCl_3_), δ (ppm); 4.70 (dd, *J* = 8.2 Hz; 2.1, 1H, =CH_2_); 4.80 (s, 2H, H_2_); 4.98 (dd, *J* = 16.0 Hz, 2.0 Hz, 1H, =CH_2_); 6,97 (t, *J* = 1.3 Hz, 1H, CH); 7,11 (t, *J* = 1.3, 1H, CH), 7.25 (dd, *J* = 16.0 Hz, 8.2 Hz, 1H, =CH); 7.57 (s, 1H, CH).

#### 3.5.3. 1-(2-Oxo-2-(vinyloxy)ethyl)-3-(4-sulfobutyl)-1H-imidazol-3-ium chloride (**11**)

Vinyl 2-(1H-imidazol-1-yl)acetate (**9**) (2.50 g, 16.00 mmol) and THF (100 mL) were placed in a 250 mL round-bottomed flask. The bath temperature was adjusted to 80 °C and stirred until the substrate had completely dissolved. At this time, 1,4-butanosultone (2.30 g, 16.00 mmol) was added dropwise. The reaction was carried out for 3 h. The reaction mixture was filtered and the solid was dried in vacuo. In the next step, the weighed (0.82 g, 10 mmol) precipitate was dissolved in THF (70 mL). The bath temperature was set to 60 °C. After a few minutes (0.84 mL, 10 mmol), concentrated hydrochloric acid was added dropwise. The reaction was stirred all night. After this time, THF was decanted and the resulting ionic liquid was dried in vacuo to give 0.96 g (19%) of pale-yellow oil.

IR (ATR, cm^−1^); 3370 (w), 3126 (w), 2921 (w), 2851 (w), 1660 (s, C=O stretching), 1632 (s), 1465 (w), 1160 (s), 1093 (s), 1038 (s)—Figure S8.

^1^H-NMR (700 MHz, CDCl_3_), δ (ppm); 1.36 (m, 2H), 1.66 (m, 2H), 2.57 (m, 2H), 3.88 (m, 2H), 4.76 (dd, *J* = 8.2 Hz; 2.1, 1H, =CH_2_); 4.82 (s, 2H, H_2_); 4.98 (dd, *J* = 16.0 Hz, 2.0 Hz, 1H, =CH_2_); 6.82 (t, *J* = 1.3 Hz, 1H, CH); 7.02 (t, *J* = 1.3, 1H, CH), 7.48 (dd, *J* = 16.0 Hz, 8.2 Hz, 1H, =CH); 7.82 (s, 1H, CH).

#### 3.5.4. Polymer VI (PVA-ImSO_3_H) (**12**)

Polymerization of 1-(2-oxo-2-(vinyloxy)ethyl)-3-(4-sulfobutyl)-1H-imidazol-3-ium chloride

1-(2-Oxo-2-(vinyloxy)ethyl)-3-(4-sulfobutyl)-1H-imidazol-3-ium chloride (**11**) (3.24 g, 10.0 mmol), azobisisobutyronitrile (AIBN) (0.18 g, 1.00 mmol) and toluene (20 mL) were placed in a pressure vessel. The reaction was carried out at 100 °C for 3 h. After this time, the separated precipitate was filtered off and dried to give 0.81 g (25%) of yellowish solid.

IR (ATR, cm^−1^); 3436 (w), 2928 (w), 2869 (s), 1673, 1606 (s, C=O stretching), 1420 (s), 1352 (s), 1317 (w, S=O stretching), 1185 (s), 1116 (s), 1051 (s)—Figure S9.

#### 3.5.5. Polymer VII (PVA-Im) (**13**)

Polymerization of vinyl 2-(1H-imidazol-1-yl)acetate

Vinyl 2-(1H-imidazol-1-yl)acetate (**9**) (2.00 g, 13.1 mmol), AIBN (0.2 g, 1.2 mmol) and toluene (20 mL) were placed in a pressure vessel. The reaction was carried out at 100 °C for 3 h. After this time, the separated precipitate was filtered off and dried to give 0.59 g (30%) of pale-yellow solid.

IR (ATR, cm^−1^): 2983 (m), 1732 (s, C=O stretching), 1446 (s), 1351 (s), 1238 (s), 1169(s)—Figure S10.

#### 3.5.6. Copolymer I (Co-NVIm-VAc) (**16**)

Copolymerization of *N*-vinylimidazole and vinyl acetate.

*N*-vinylimidazole (14) (2.04 g, 21.5 mmol), vinyl acetate (**15**) (1.85 g, 21.5 mmol), AIBN (0.4 g, 2.4 mmol), and toluene (20 mL) were placed in a pressure vessel. The reaction was carried out at 100 °C for 3 h. After this time, the separated precipitate was filtered off and dried to give 2.78 g (71%) of pale-yellow powder.

IR (ATR, cm^−1^); 3055 (m), 2961 (m), 2935 (m), 2873 (m), 1744 (s, C=O stretching), 1694 (w), 1464 (m), 1222 (s), 1165 (s), 1015 (s)—Figure S11.

#### 3.5.7. Copolymer II (Co-NVIm-PVA) (**17**)

Hydrolysis of copolymer I 

Copolymer I (**16**) (1.00 g, mmol) was placed in a 25 mL round bottom flask, and a saturated solution of sodium hydroxide in methanol (5 mL) was added. The mixture was stirred for 30 min, and then the product was filtered through a Büchner funnel washing twice with methanol. A white solid 0.64 g (72%) was obtained.

IR (ATR, cm^−1^); 3387 (m), 2937 (w), 2830 (w), 1660 (m), 1349 (m), 1188 (s), 1168 (s), 1029 (s)—Figure S12.

^1^H-NMR (700 MHz, MeOD), δ (ppm); 3.23 (s); 3.29 (q, *J* = 1.7); 3.33 (s); 3.43 (s); 5.15 (s).

Anal. Calcd for C_7_H_10_N_2_O: C, 60.85; H, 7.30; N, 20.27. Found: C, 61.24; H, 7.06; N, 21.01.

### 3.6. Reaction of N-Methylimidazole with Perfluorinated Sultones

#### 3.6.1. IL I (**22**)

1,2,2-trifluoro-2-hydroxy-1-trifluoromethylethanesulfonic acid sultone (**20**) (2.30 g, 10 mmol) was placed in a 50 mL round bottom flask followed by the addition of toluene (5 mL). The reaction flask was heated at 80 °C. Subsequently, 1-methylimidazole (0.82 g, 10 mmol) dissolved in toluene (5 mL) was added dropwise, stirred, and heated for 48 h. After this time, a white precipitate formed in the flask, which was filtered off, washed with toluene and dried in vacuo to give 1.97 g (90%) oil. To a 50 mL round-bottomed flask the oil (2.19 g, 10 mmol) was added toluene, 10 mL, followed by concentrated hydrochloric acid (0.84 mL, 10 mmol). The mixture was stirred at 60 °C for 24 h. A viscous oily liquid was formed after evaporation of solvent, 1.43 g (46%). 

IR (ATR, cm^−1^); 3126 (w), 3072 (w), 1586 (w), 1546 (w), 1285 (w), 1214 (s), 1175 (s), 1142 (s), 1011 (s)—Figure S14.

^1^H-NMR (400 MHz, CDCl_3_), δ (ppm); 3.52 (s, 3H), 6.71 (t, *J* = 1.4 Hz, 1H, CH); 7.11 (t, *J* = 1.4, 1H, CH), 7.64 (s, 1H, CH). 

^19^F-NMR (700 MHz, CDCl_3_, relative to C_6_F_6_), δ (ppm); −75.1 (3 F), −83.5 (1 F), −89.6 (1 F), −153.8 (1F)—Figure S15.

#### 3.6.2. IL II (**23**)

Reaction with 1-(nonafluorobutyl)trifluoroethanesultone

1-(Nonafluorobutyl)trifluoroethanesultone (21) (3.80 g, 10 mmol) was placed in a 50 mL round bottom flask followed by the addition of toluene (5 mL). The reaction flask was heated at 80 °C. Then, 1-methylimidazole (19) (0.82 g, 10 mmol) dissolved in toluene (5 mL) was added dropwise, stirred, and heated for 48 h. After this time, a white precipitate formed in the flask, which was filtered off, washed with toluene and dried in vacuo to give 1.97 g (90%) oil. To a 50 mL round-bottomed flask the oil (2.19 g, 10 mmol) was added in toluene, 10 mL followed by concentrated hydrochloric acid (0.84 mL, 10 mmol). The mixture was stirred at 60 °C for 24 h. A viscous oily liquid was formed after evaporation of solvent, 2.40 g (52%).

IR (ATR, cm^−1^); 3127 (w), 2959 (w), 1586 (w), 1547 (w), 1282 (w), 1211 (s), 1178 (s), 1037 (s)—Figure S16.

^1^H-NMR (400 MHz, CDCl_3_), δ (ppm); 3.54 (s, 3H), 6.76 (t, *J* = 1.4 Hz, 1H, CH); 7.18 (t, *J* = 1.4, 1H, CH), 7.72 (s, 1H, CH). 

^19^F-NMR (700 MHz, CDCl_3_, relative to C_6_F_6_), δ (ppm); δ (ppm); −82.0 (2 F), −83.0 (1 F), −86.2 (2 F), −117.7 (2 F), −121.6 (2 F), 127.6 (2 F), −152.1 (1F)—Figure S17.

### 3.7. Reaction of Copolymer II (Co-NVIm-PVA) with Sultones

#### 3.7.1. Copolymer III (Co-NVIm-PVA-1,4) (**18**)

Reaction with 1,4-Butanosultone

Copolymer II (17) (0.44 g, 10 mmol) and *N*-methylpyrrolidone (10 mL) were placed in a 25 mL two-necked flask. The dissolution of polymer was carried out at 120 °C under a nitrogen atmosphere. After one hour, the temperature was lowered to 50 °C and a solution of 1,4-butanesultone (0.14 g, 1.00 mmol) in NMP (2 mL) was added. Then, a solution of potassium *t*-butoxide (0.12 g, 1.00 mmol) in NMP (2 mL) was slowly added dropwise. The reaction was carried out for 1 h at room temperature. The flask residue was transferred to a beaker with 100 mL acetone. A white polymer precipitated in the beaker, which was filtered off and dried in vacuo. Then, the product was suspended in acetonitrile (20 mL) and concentrated hydrochloric acid was added (0.84 mL, 10 mmol). The mixture was stirred for 48 h at room temperature under nitrogen atmosphere. The precipitate was filtered off. Moreover, it was washed with acetonitrile and dried in vacuo to give 0.19 g (32%) of brownish powder.

IR (ATR, cm^−1^); 3450 (w), 3126 (w), 3067 (w), 2962 (w), 1649 (w), 1315 (w), 1212 (s), 1174 (s), 1142 (s), 1011 (s)—Figure S13.

#### 3.7.2. Copolymer IV (Co-NVIm-PVA-6F) (**24**)

Reaction with 1,2,2-Trifluoro-2-hydroxy-1-trifluoromethylethanesulfonic acid sultone

Copolymer II (**17**) (0.44 g, 10 mmol) and *N*-methylpyrrolidone (10 mL) were placed in a 50 mL two-necked flask. The dissolution of copolymer II was carried out at 120 °C under a nitrogen atmosphere. After one hour, the temperature was lowered to 50 °C and a solution of 1,2,2-trifluoro-2-hydroxy-1-trifluoromethylethanesulfonic acid sultone (20) (0.24 g, 2.0 mmol) in NMP (3.5 mL) was added. Then, a solution of potassium *t*-butoxide (0.24 g, 2.0 mmol) in NMP (3.5 mL) was slowly added dropwise. The reaction was carried out for 1 h at room temperature. The flask residue was transferred to a beaker with 100 mL acetone. A white polymer precipitated in the beaker, which was filtered off and dried in vacuo. Then, the product was suspended in acetonitrile (20 mL), and concentrated hydrochloric acid was added (0.84 mL, 10 mmol). The mixture was stirred for 48 h at room temperature under nitrogen atmosphere. The precipitate was filtered off, washed with acetonitrile, and dried in vacuo to give 0.46 g (67%) of brownish powder.

IR (ATR, cm^−1^); 3126 (w), 1587 (w), 1281 (s), 1183 (s), 1078 (s), 1039 (s)—Figure S18.

#### 3.7.3. Copolymer V (Co-NVIm-PVA-12F) (**25**)

Reaction with 1-(Nonafluorobutyl)trifluoroethanesultone

Copolymer II (**17**) (0.44 g, 10 mmol) and *N*-methylpyrrolidone (10 mL) were placed in a 50 mL two-necked flask. The dissolution of copolymer II was carried out at 120 °C under a nitrogen atmosphere. After one hour, the temperature was lowered to 50 °C and a solution of 1-(nonafluorobutyl)trifluoroethanesultone (**21**) (0.32 g, 2.0 mmol) in NMP (3.5 mL) was added. Then, a solution of potassium t-butoxide (0.24 g, 2.0 mmol) in NMP (3.5 mL) was slowly added dropwise. The reaction was carried out for 1 h at room temperature. A white polymer precipitated in the beaker, which was filtered off and dried in vacuo. Then, the product was suspended in acetonitrile (20 mL), and concentrated hydrochloric acid was added (0.84 mL, 10 mmol). The mixture was stirred for 48 h at room temperature under nitrogen atmosphere. The precipitate was filtered off, washed with acetonitrile, and dried in vacuo to give 0.54 g (62%) of gray powder.

IR (ATR, cm^−1^); 3126 (w), 3070 (w), 1586 (w), 1546 (w), 1285 (m), 1213 (s), 1175 (s), 1142 (s), 1030 (s)—Figure S19.

## 4. Conclusions

Modification of PVA by forming ester groups with free hydroxyl groups encounters difficulties related to its solubility or lack of organic solvents. Therefore, the reactions take place with low efficiency, and this method of modification has numerous disadvantages, as shown in this paper. Thus, to overcome these disadvantages, copolymers based on vinyl imidazole and vinyl acetate were obtained. They were modified with alkyl and perfluoroalkylsulfone chains to obtain PVA derivatives that contain a moiety of protic ionic liquids with a sulfonic acid group.

The presented synthesis route is a novel approach to PVA functionalization. The obtained PVA derivatives may be potentially used as an advanced polymer material for the elaboration of proton exchange membranes for medium- and high-temperature PEMFC. The developed synthesis method of chemical PVA modification is a promising approach to minimize the release of ionic liquid from the polymer matrix during the process.

## Data Availability

The data presented in this study are available on request from the corresponding author.

## Supplementary Materials

The following are available online at, Figure S1. IR spectrum of polymer I (PVA-Ac) (**2**); Figure S2. IR spectrum of polymer II (PVA-AcBr) (**3**); Figure S3. IR spectrum of polymer III (PVA-AcCl) (**4**); Figure S4. IR spectrum of polymer IV (PVA-AcIm) (**5**); Figure S5. IR spectrum of polymer V (PVA-AcIm-1,4) (**6**); Figure S6. IR spectrum of vinyl 2-iodoacetate (**8**); Figure S7. IR spectrum of vinyl 2-(1H-imidazol-1-yl)acetate (**9**); Figure S8. IR spectrum of 1-(2-Oxo-2-(vinyloxy)ethyl)-3-(4-sulfobutyl)-1H-imidazol-3-ium chloride (**11**); Figure S9. IR spectrum of polymer VI (PVA-ImSO3H) (**12**); Figure S10. IR spectrum of Polymer VII (PVA-Im) (**13**); Figure S11. IR spectrum of copolymer I (Co-NVIm-VAc) (**16**); Figure S12. IR spectrum of copolymer II (Co-NVIm-PVA) (**17**); Figure S13. IR spectrum of copolymer III (Co-NVIm-PVA-1,4) (**18**); Figure S14. IR spectrum of IL I (**22**); Figure S15. 19F NMR spectrum of IL I (**22**); Figure S16. IR spectrum of IL II (**23**); Figure S17. 19F NMR spectrum of IL II (**23**); Figure S18. IR spectrum of copolymer IV (Co-NVIm-PVA-6F) (**24**); Figure S19. IR spectrum of Copolymer V (Co-NVIm-PVA-12F) (**25**).

## Sample Availability

Samples of the compounds are not available from the authors.
