# Supplementary material for: The Synthesis of Poly(Vinyl Alcohol) Grafted with Fluorinated Protic Ionic Liquids Containing Sulfo Functional Groups
Source: Molecules. 2021 Jul 8;26(14):4158. doi: 10.3390/molecules26144158 (PMC8306934; doi:10.3390/molecules26144158)
Supplement: Supplementary file 1 [file molecules-26-04158-s001.zip › Supporting Information corrected.pdf]

Supporting Information

The synthesis of poly(vinyl alcohol) grafted with fluorinated protic ionic liquids containing sulfo functional groups

Patrycja Glińska [1], Andrzej Wolan [1, 2], Wojciech Kujawski [1,*], Edyta Rynkowska [1], and Joanna Kujawa [1]

[1] Nicolaus Copernicus University in Toruń, Faculty of Chemistry, 7 Gagarina Street, 87-100 Toruń, Poland
[2] Synthex Technologies Sp. z o.o., 7 Gagarina Street, 87-100 Toruń, Poland
* Correspondence: kujawski@chem.umk.pl

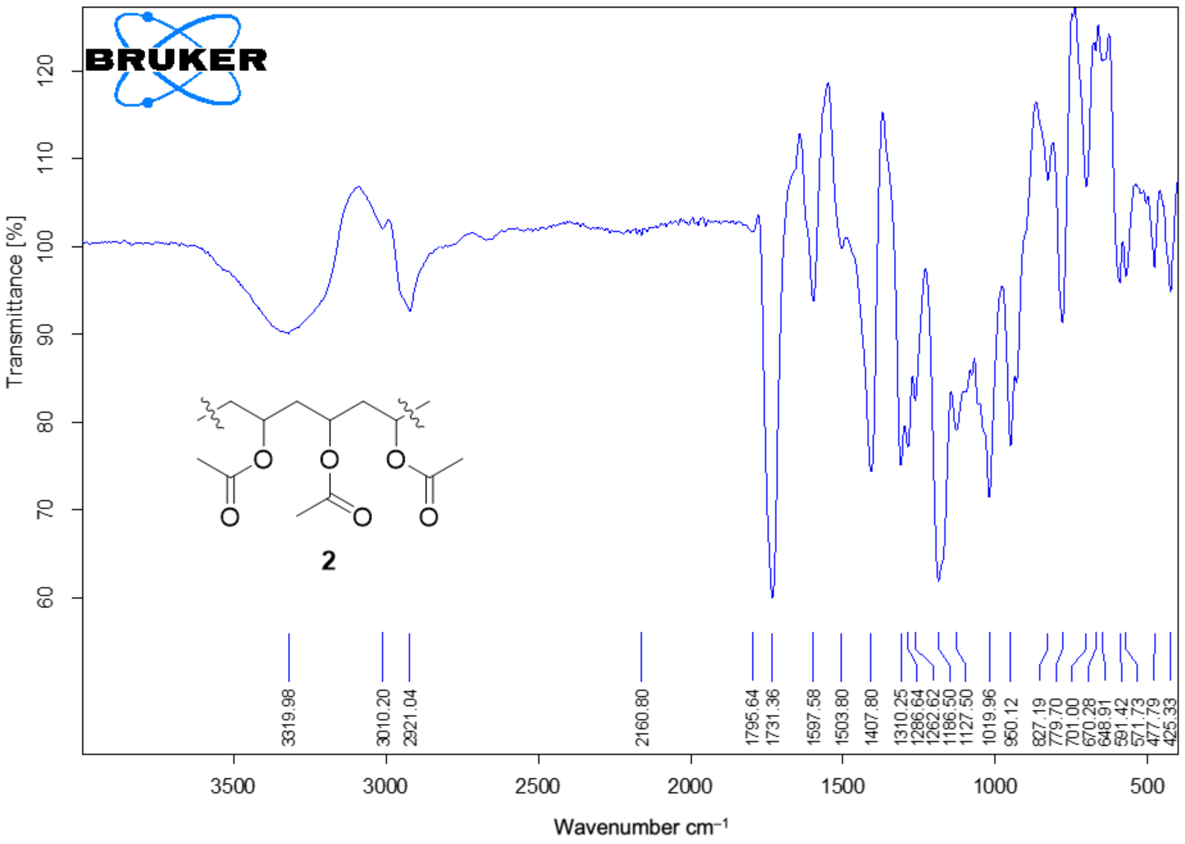

Figure S1. IR spectrum of polymer I (PVA-Ac) (**2**)

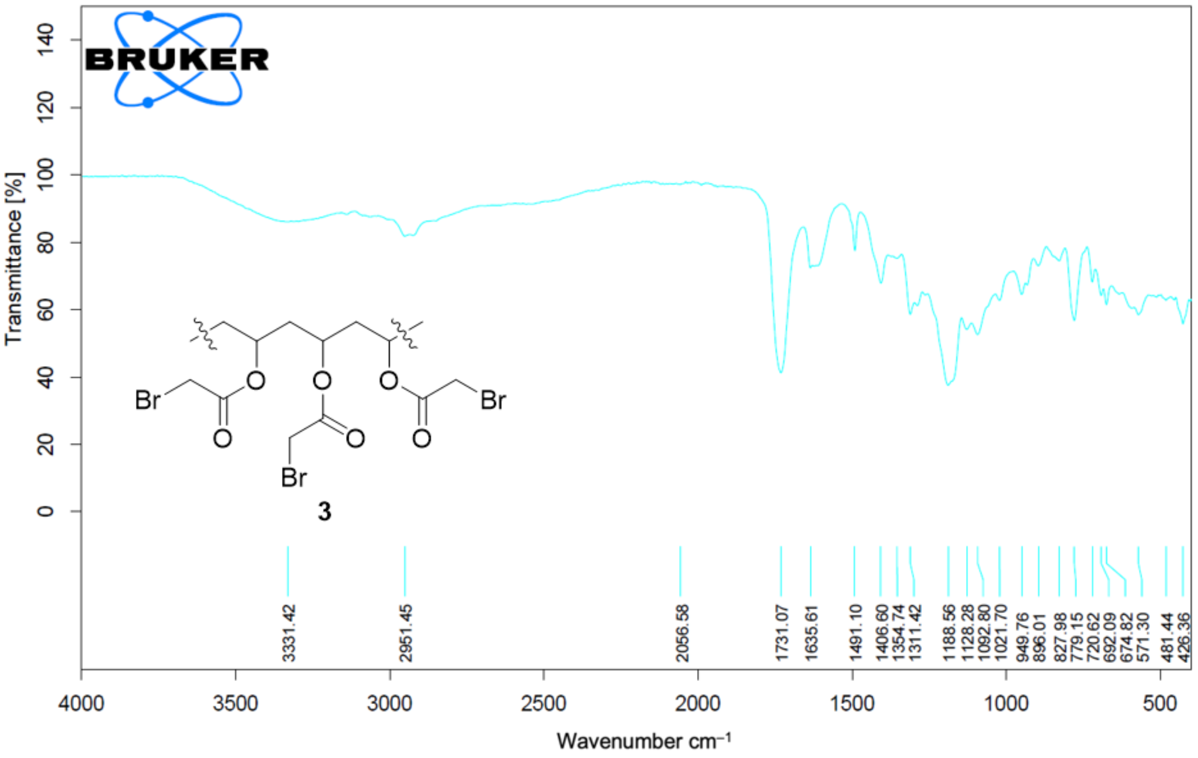

Figure S2. IR spectrum of polymer II (PVA-AcBr) (**3**)

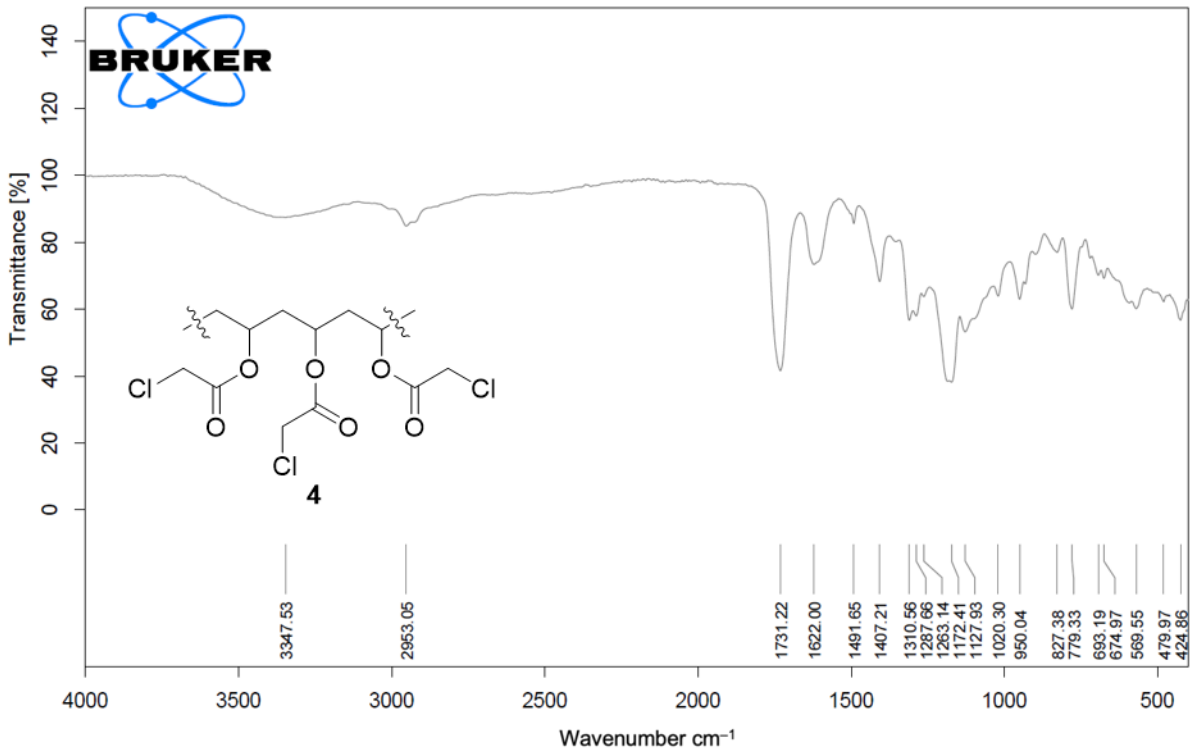

Figure S3. IR spectrum of polymer III (PVA-AcCl) (**4**)

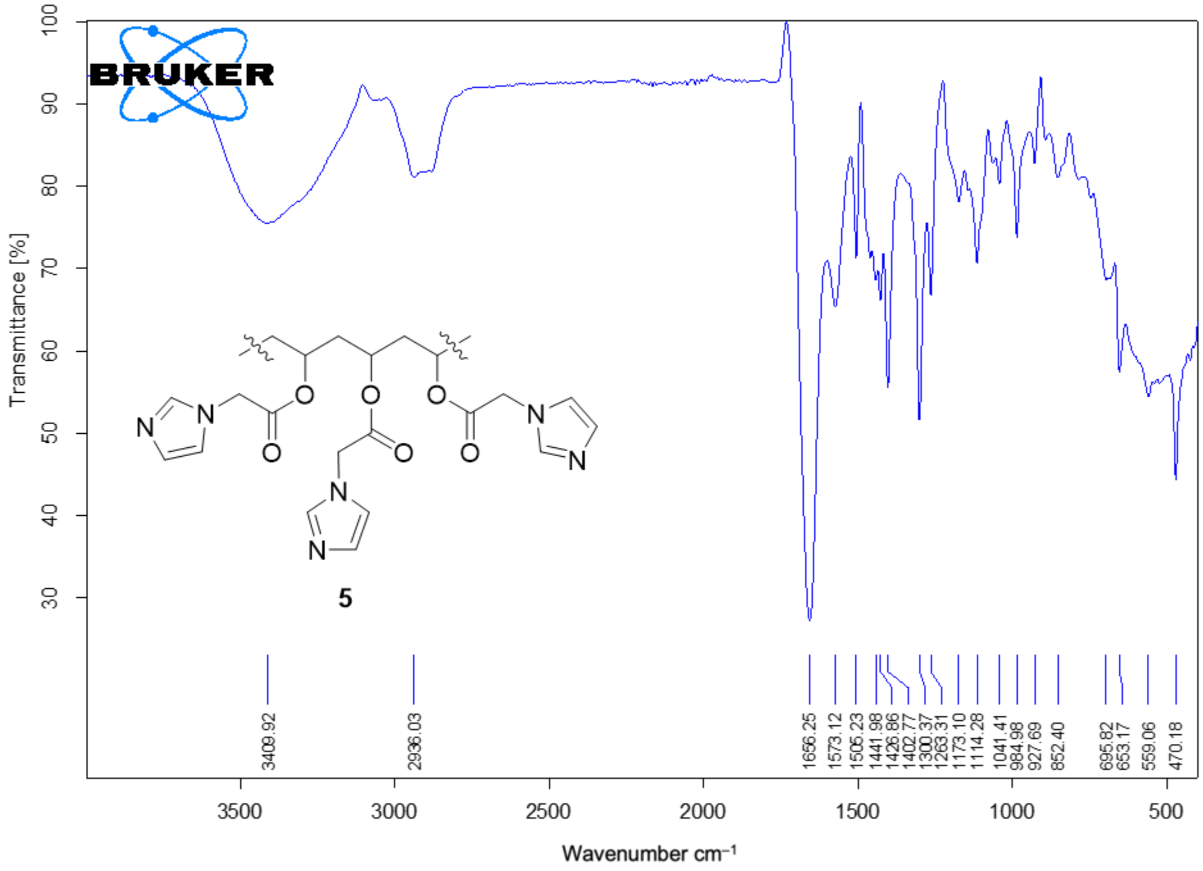

Figure S4. IR spectrum of polymer IV (PVA-AcIm) (**5**)

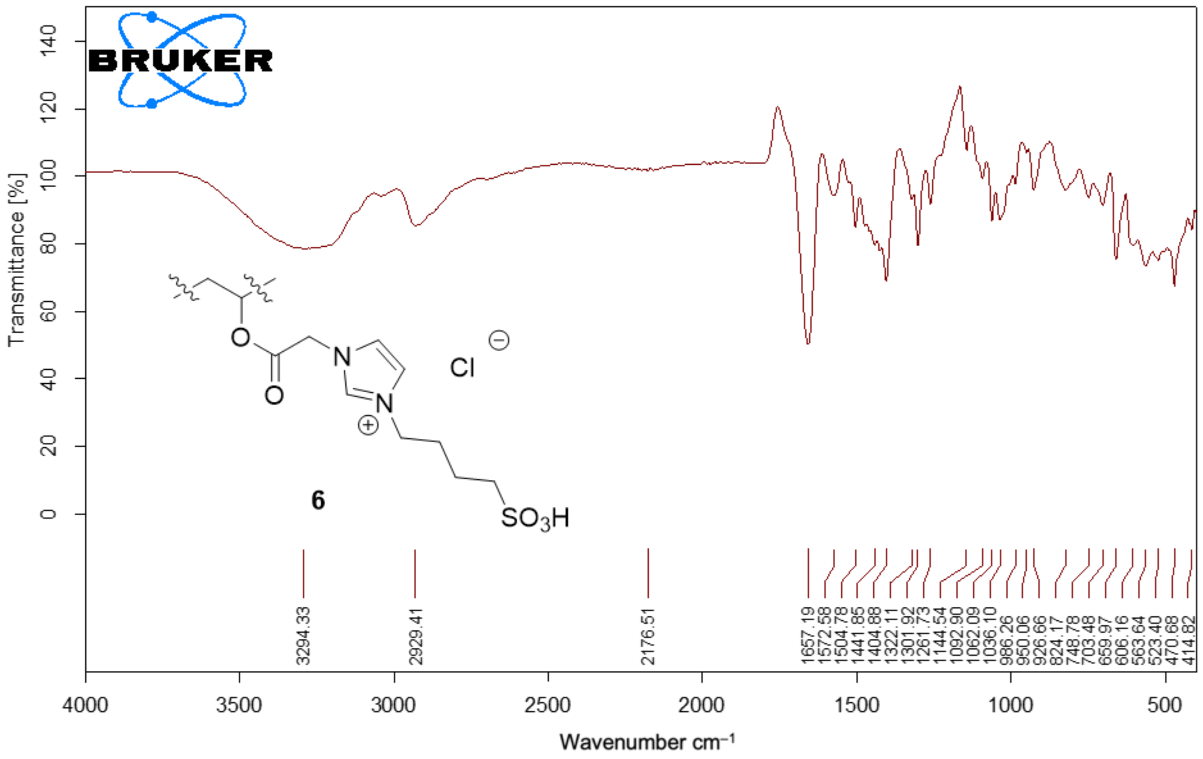

Figure S5. IR spectrum of polymer V (PVA-AcIm-1,4) (**6**)

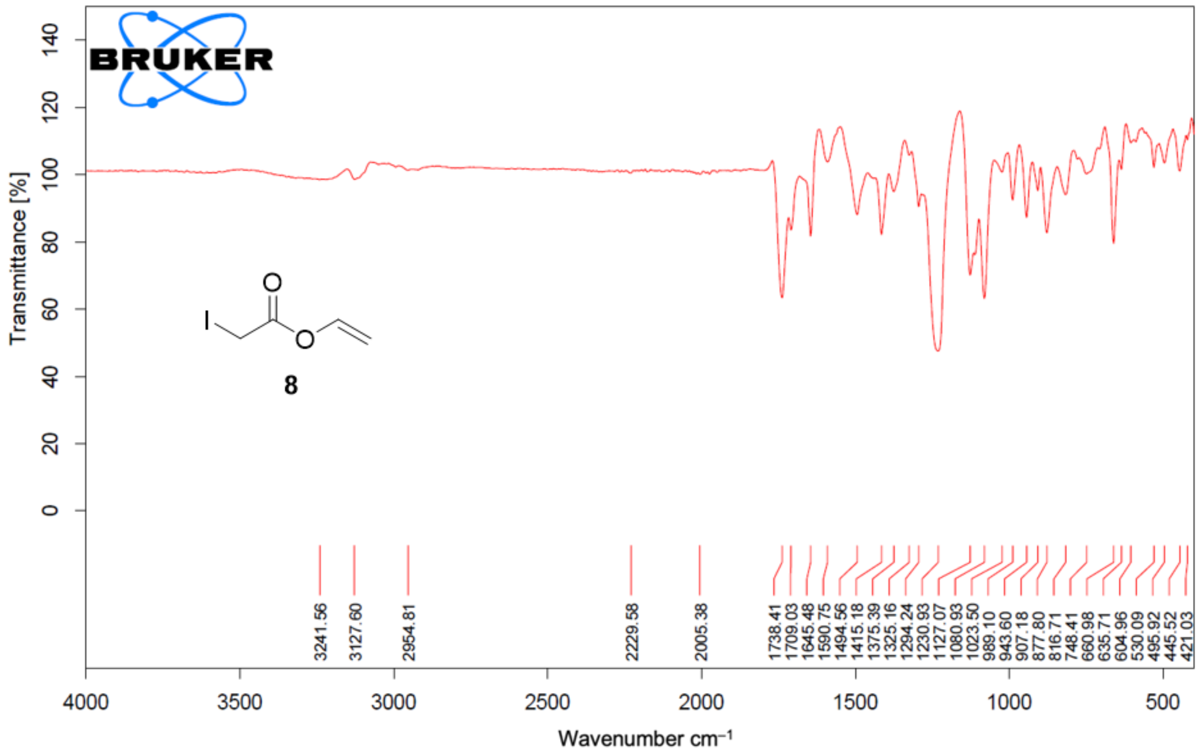

Figure S6. IR spectrum of vinyl 2-iodoacetate (**8**)

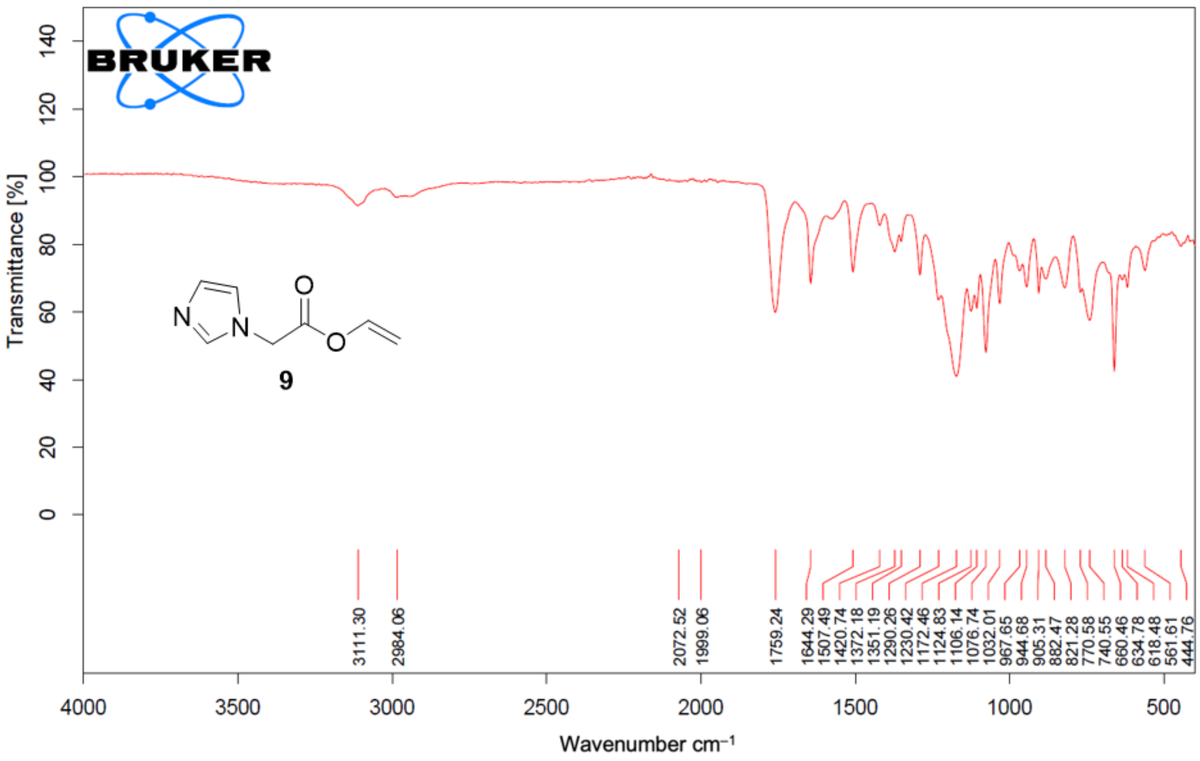

Figure S7. IR spectrum of vinyl 2-(1H-imidazol-1-yl)acetate (**9**)

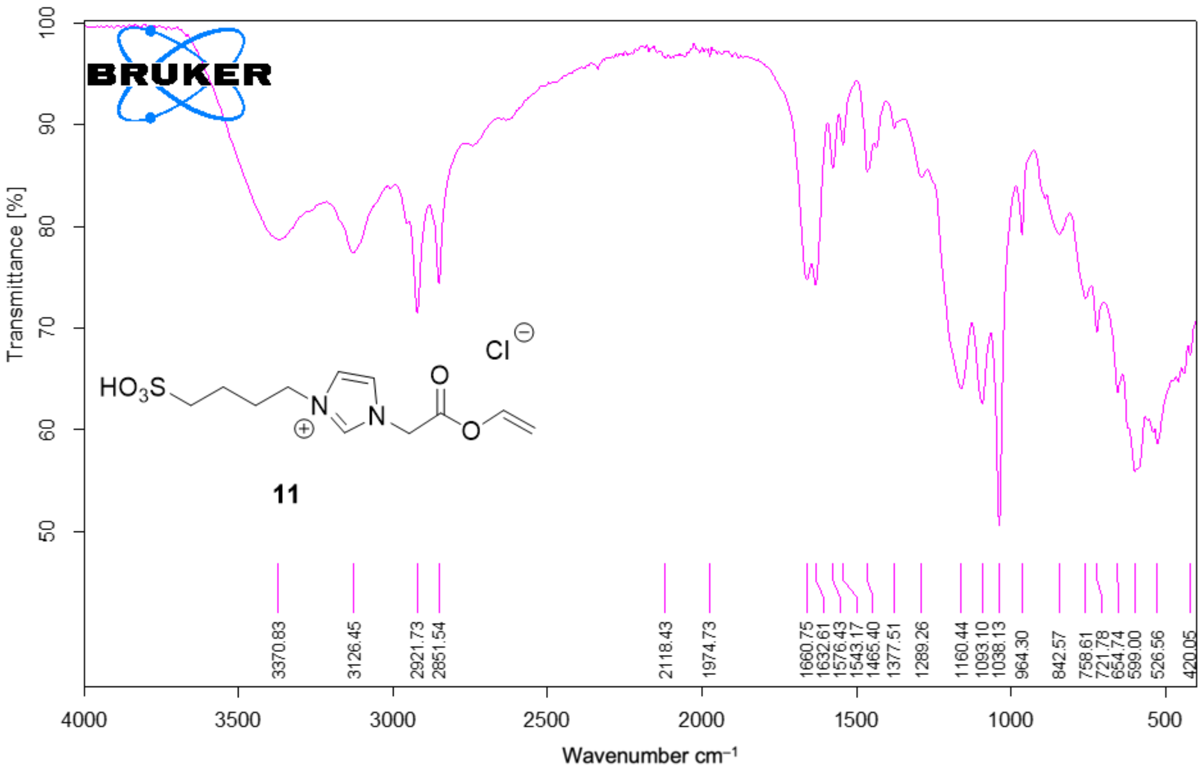

Figure S8. IR spectrum of 1-(2-Oxo-2-(vinyloxy)ethyl)-3-(4-sulfobutyl)-1H-imidazol-3-ium chloride (**11**)

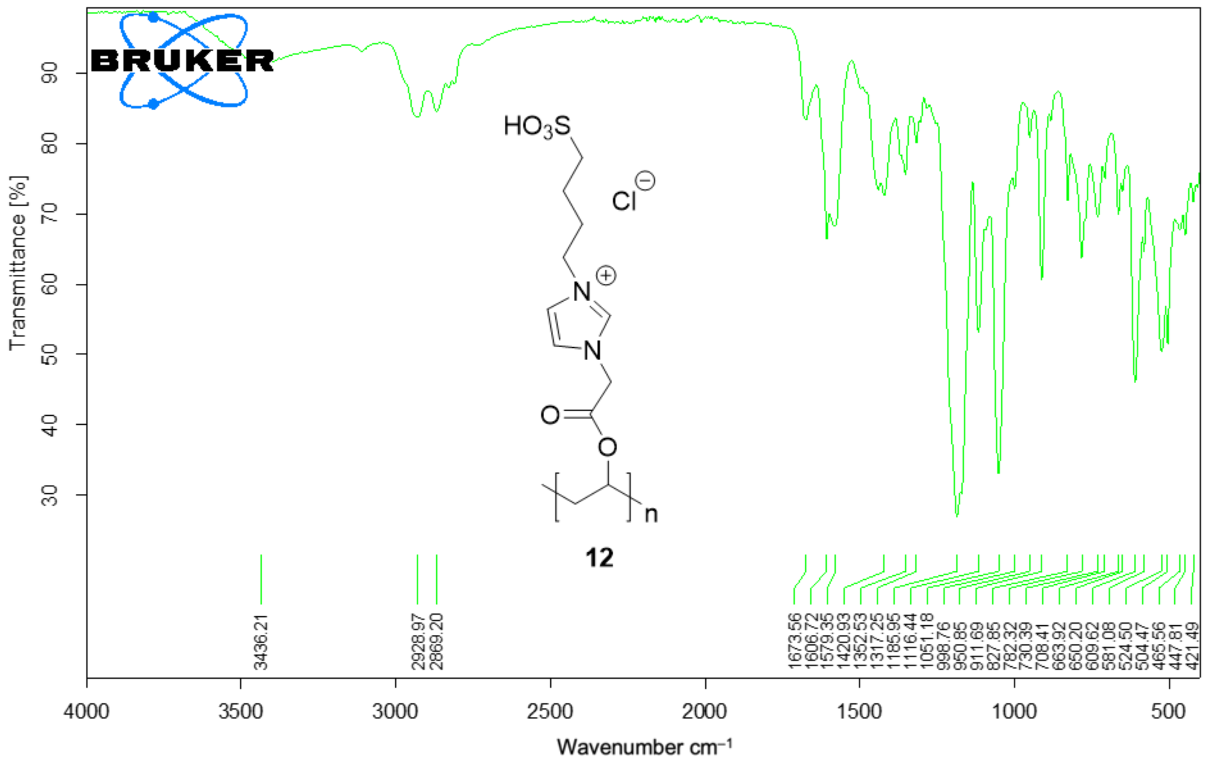

Figure S9. IR spectrum of polymer VI (PVA-ImSO₃H) (**12**)

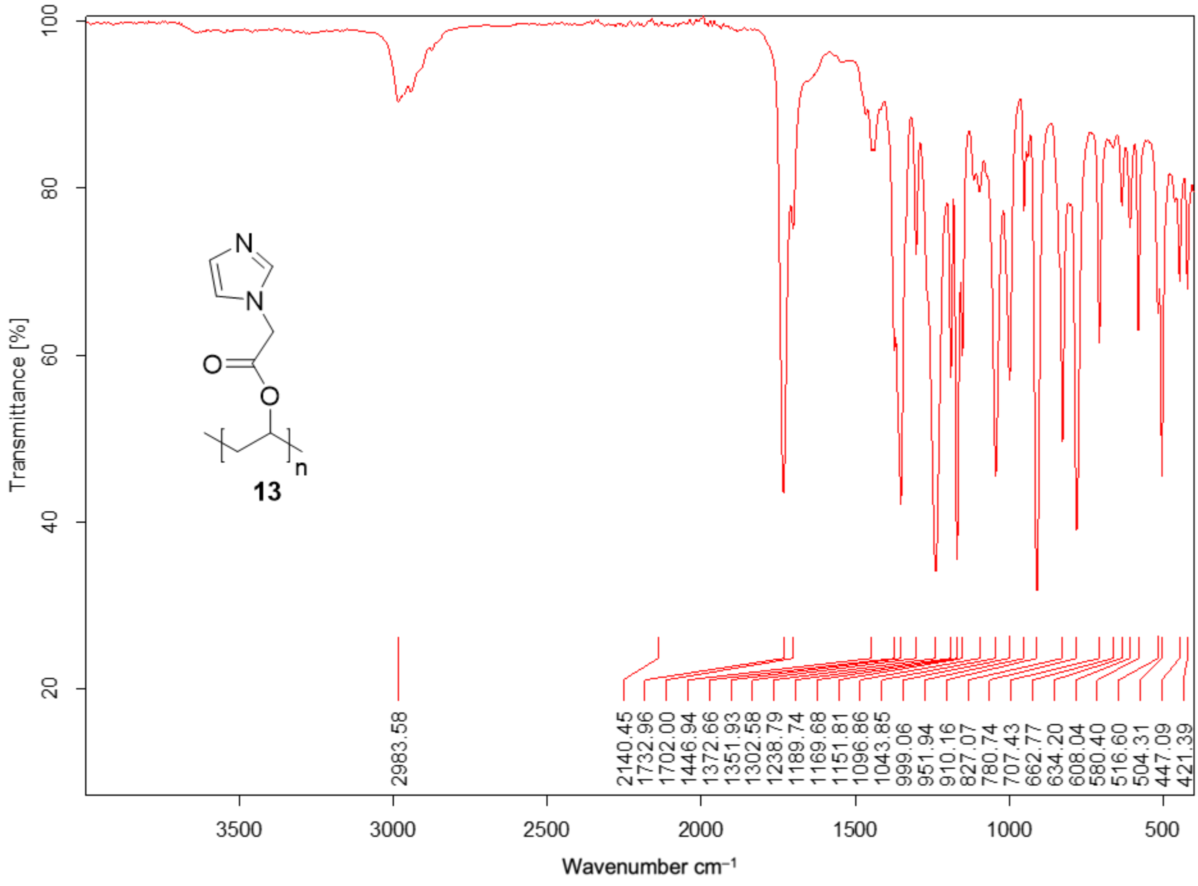

Figure S10. IR spectrum of Polymer VII (PVA-Im) (**13**)

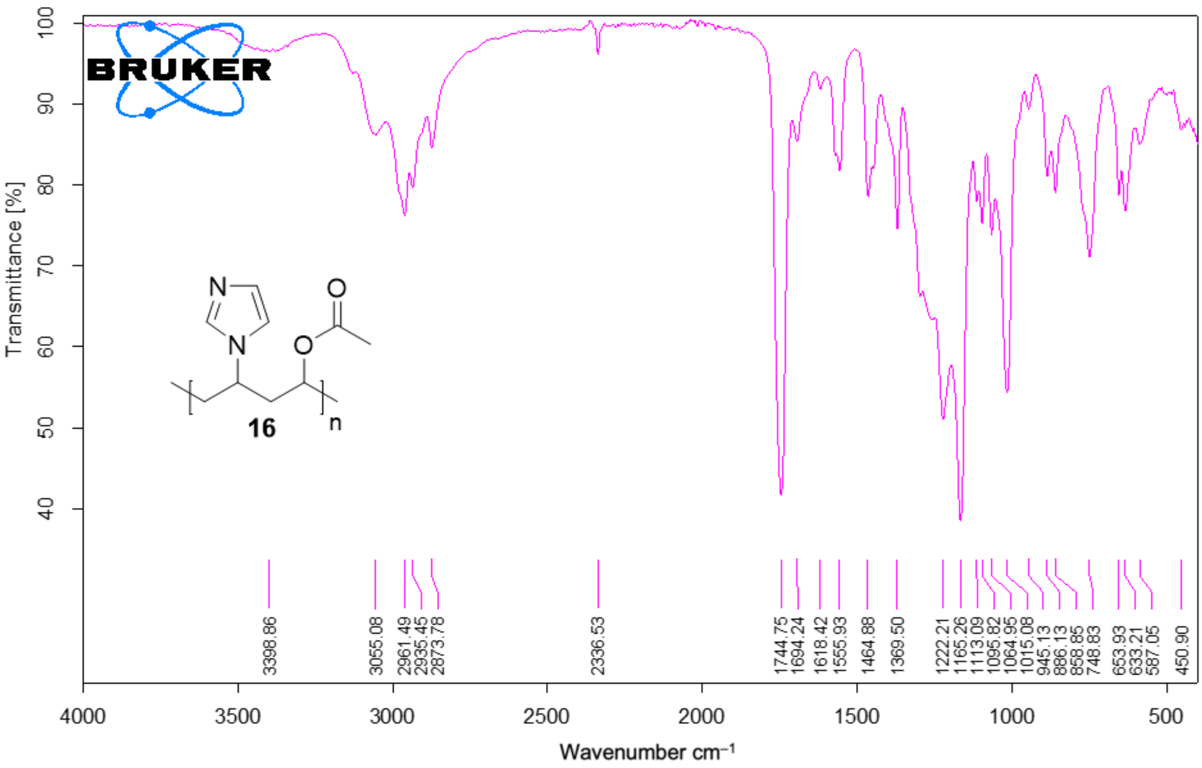

Figure S11. IR spectrum of copolymer I (Co-NVIm-VAc) (**16**)

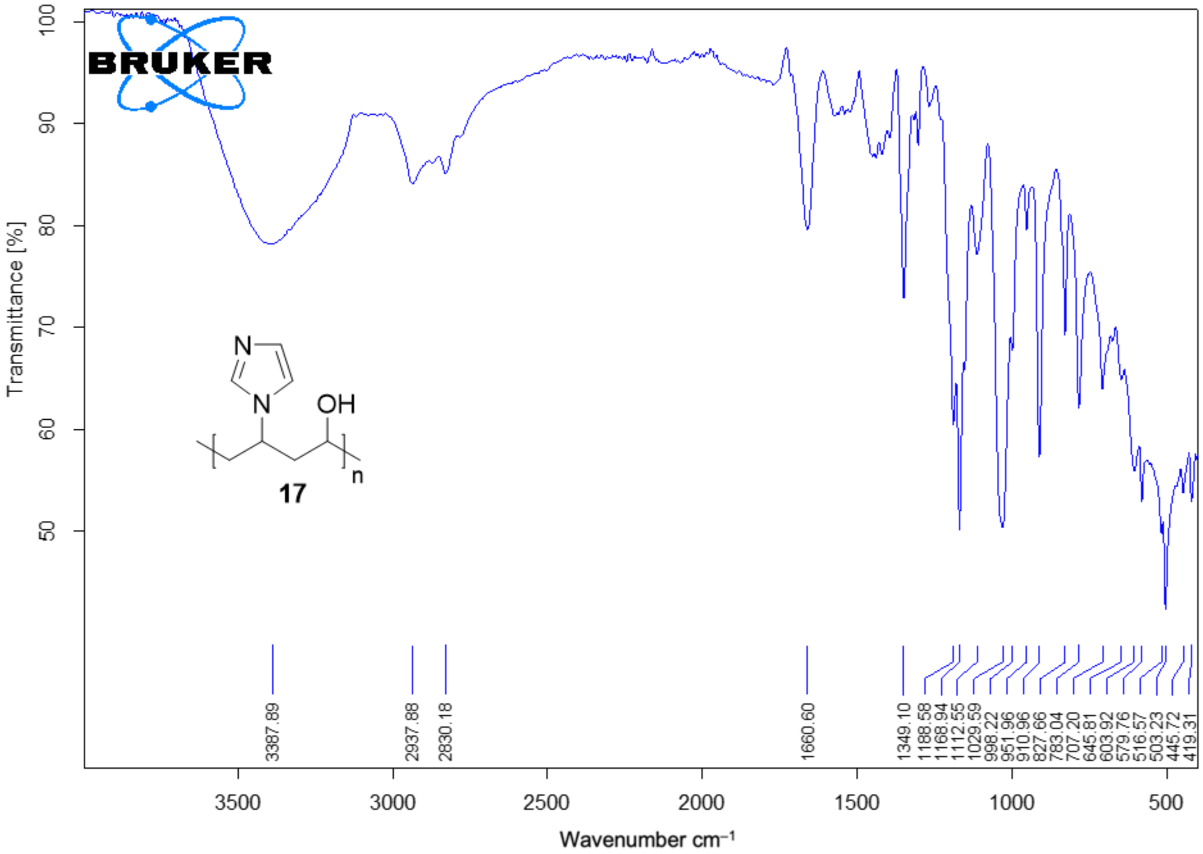

Figure S12. IR spectrum of copolymer II (Co-NVIm-PVA) (**17**)

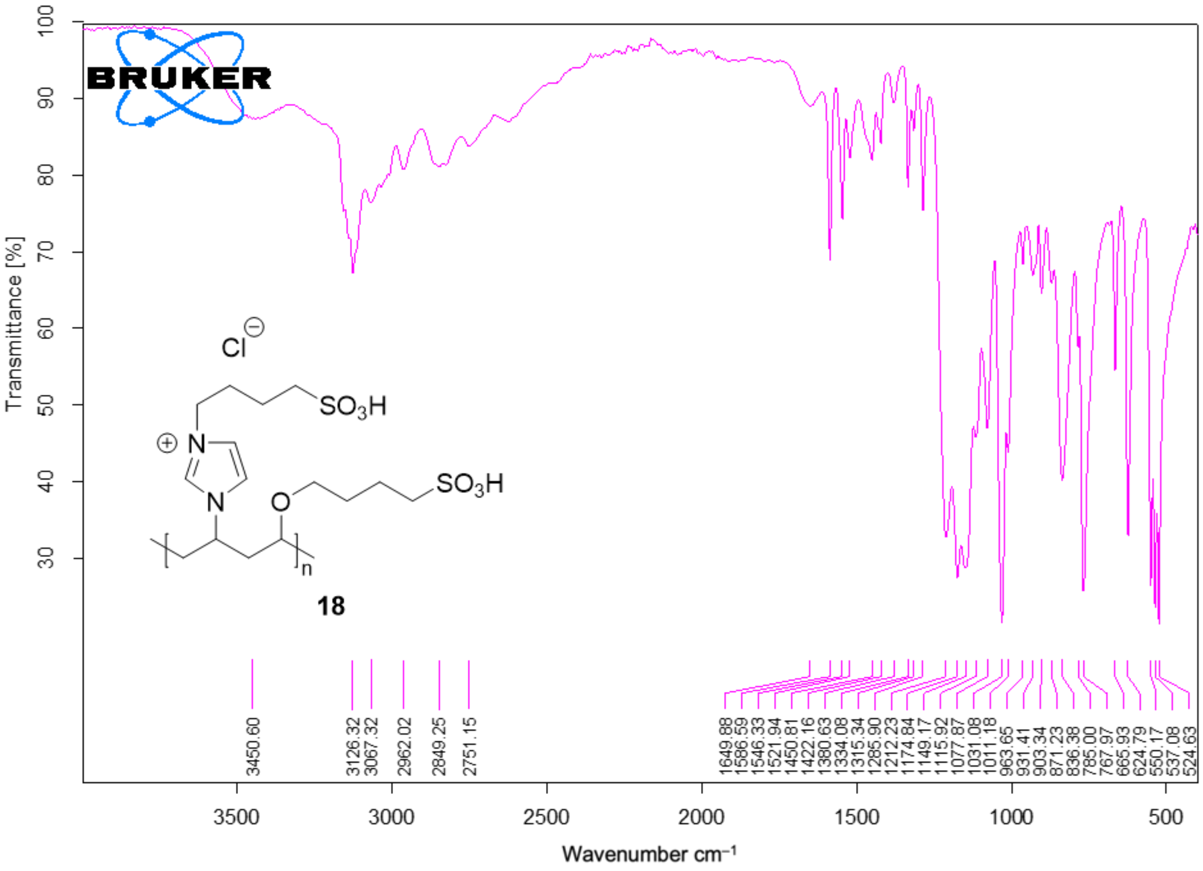

Figure S13. IR spectrum of copolymer III (Co-NVIm-PVA-1,4) (**18**)

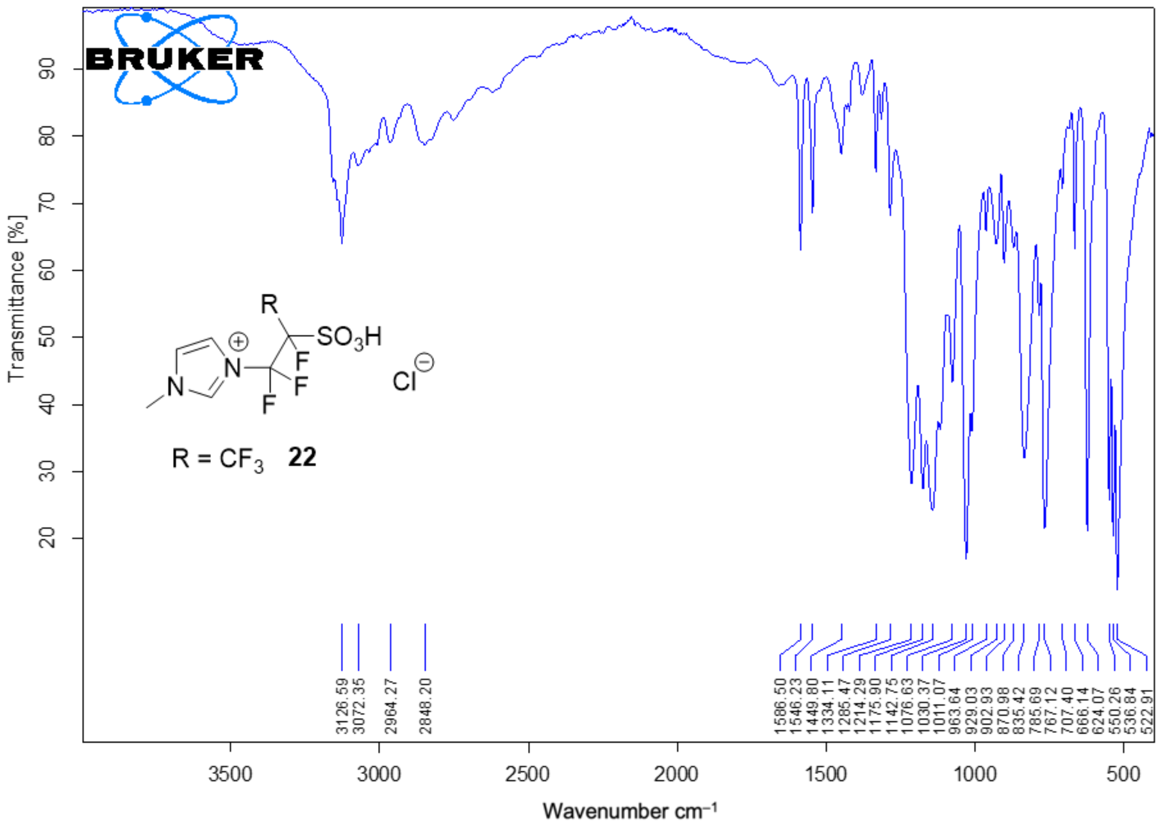

Figure S14. IR spectrum of IL I (**22**)

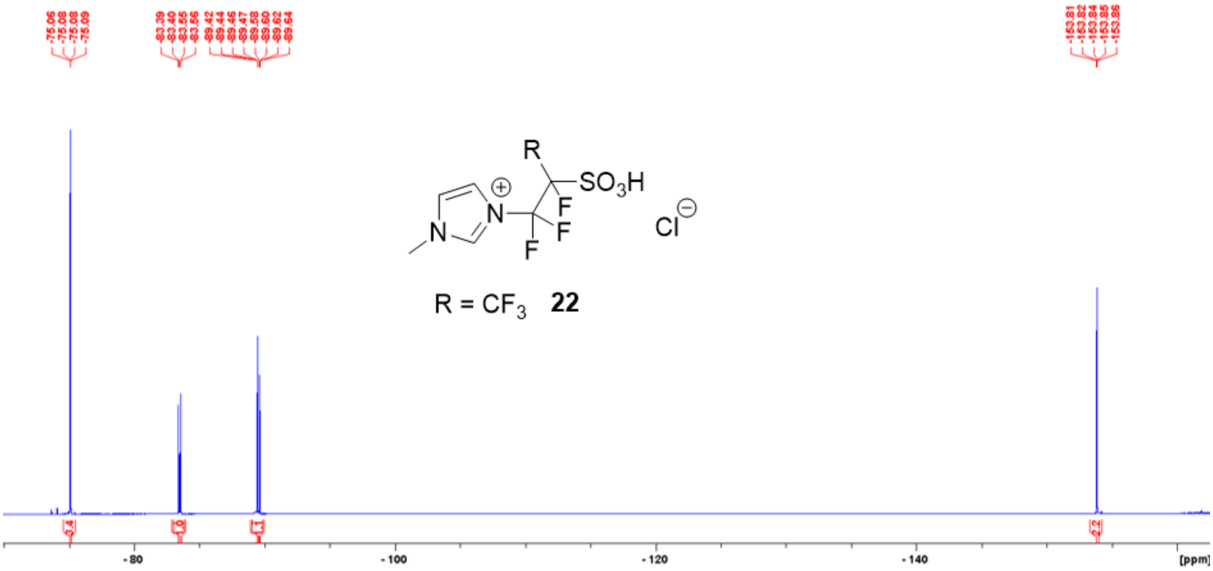

Figure S15. 19F NMR spectrum of IL I (**22**)

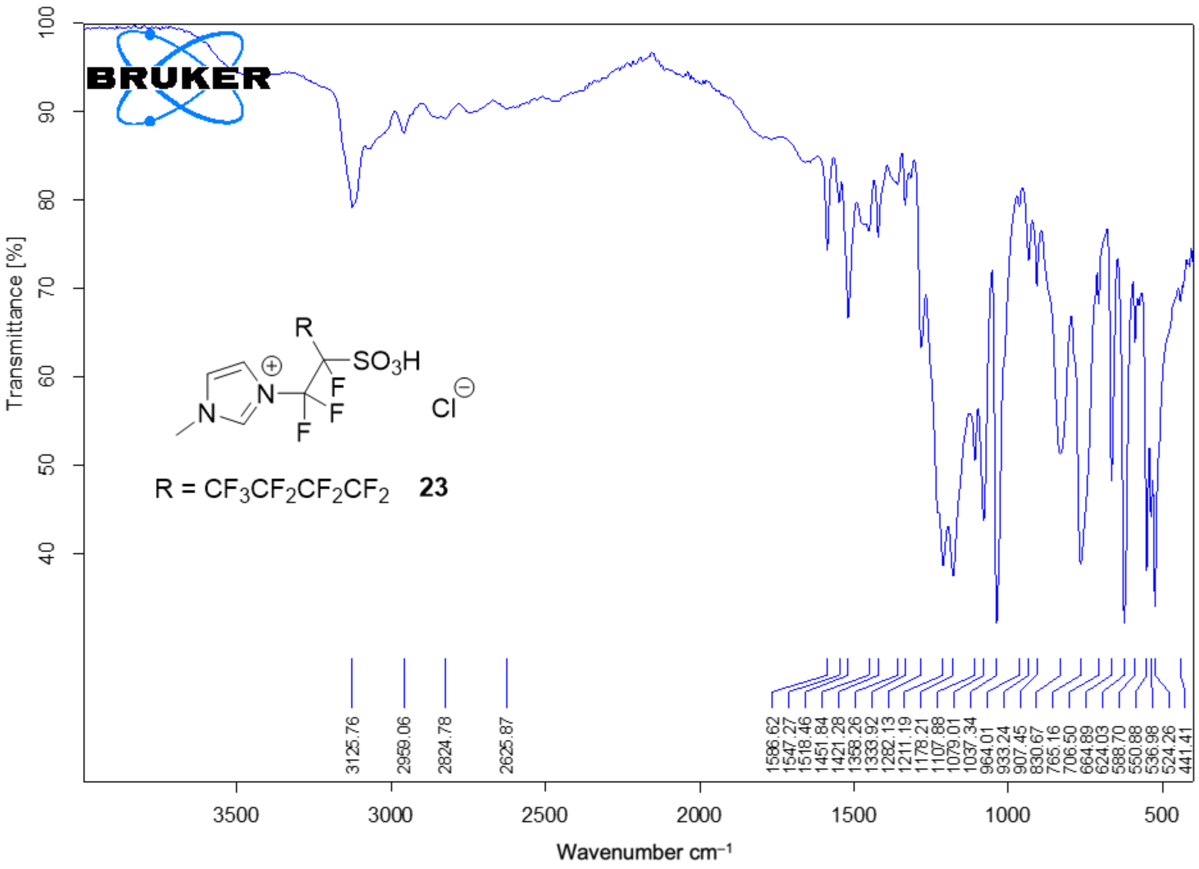

Figure S16. IR spectrum of IL II (**23**)

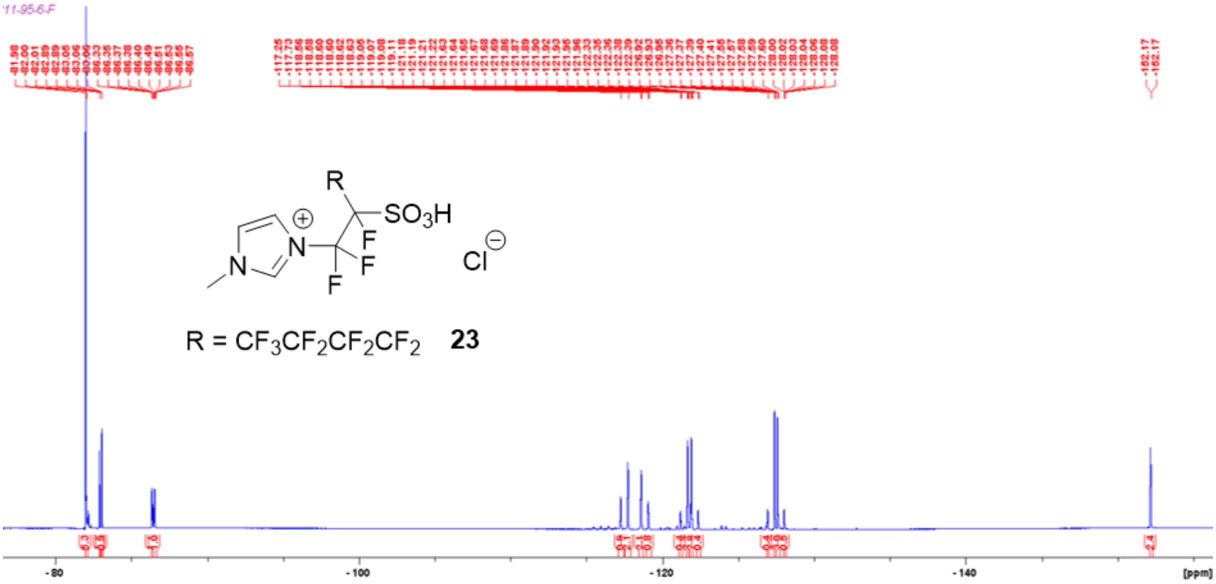

Figure S17. 19F NMR spectrum of IL II (**23**)

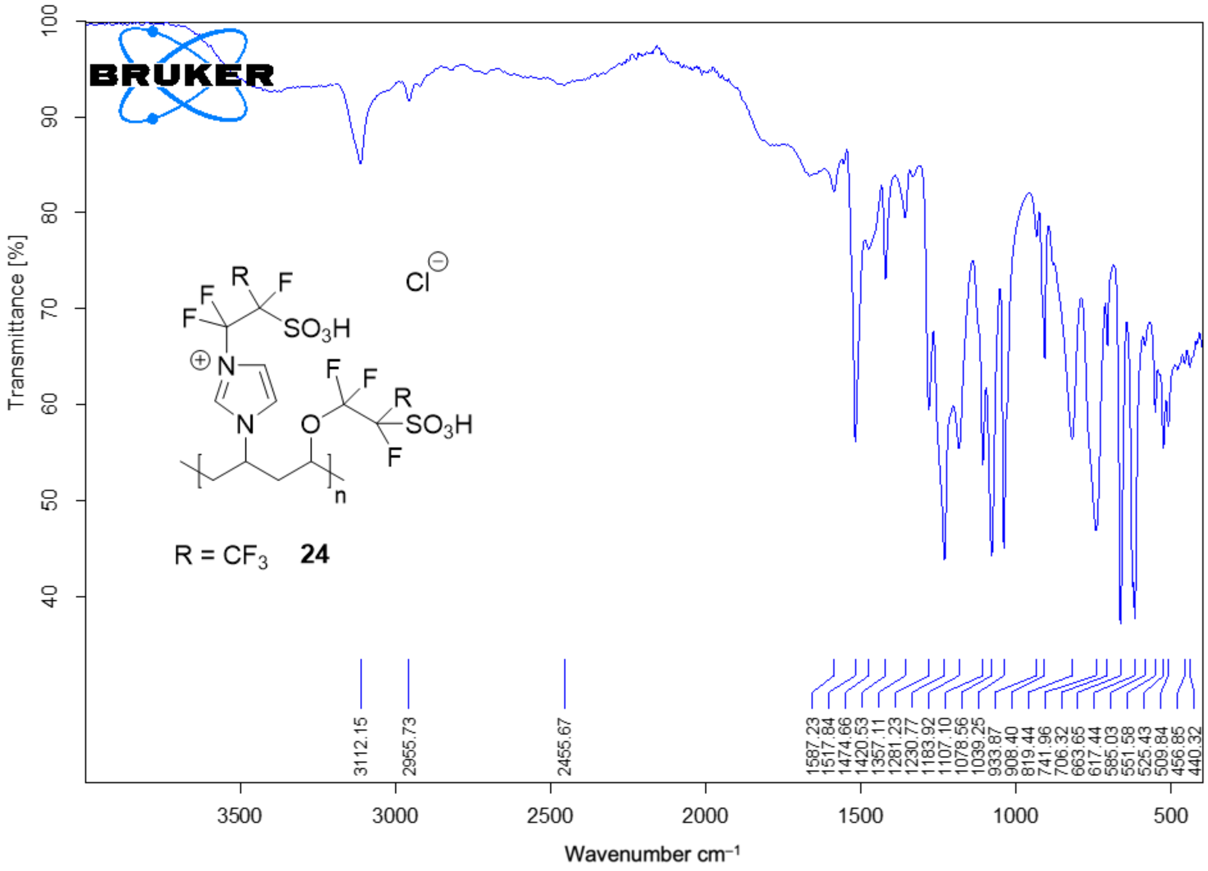

Figure S18. IR spectrum of copolymer IV (Co-NVIm-PVA-6F) (**24**)

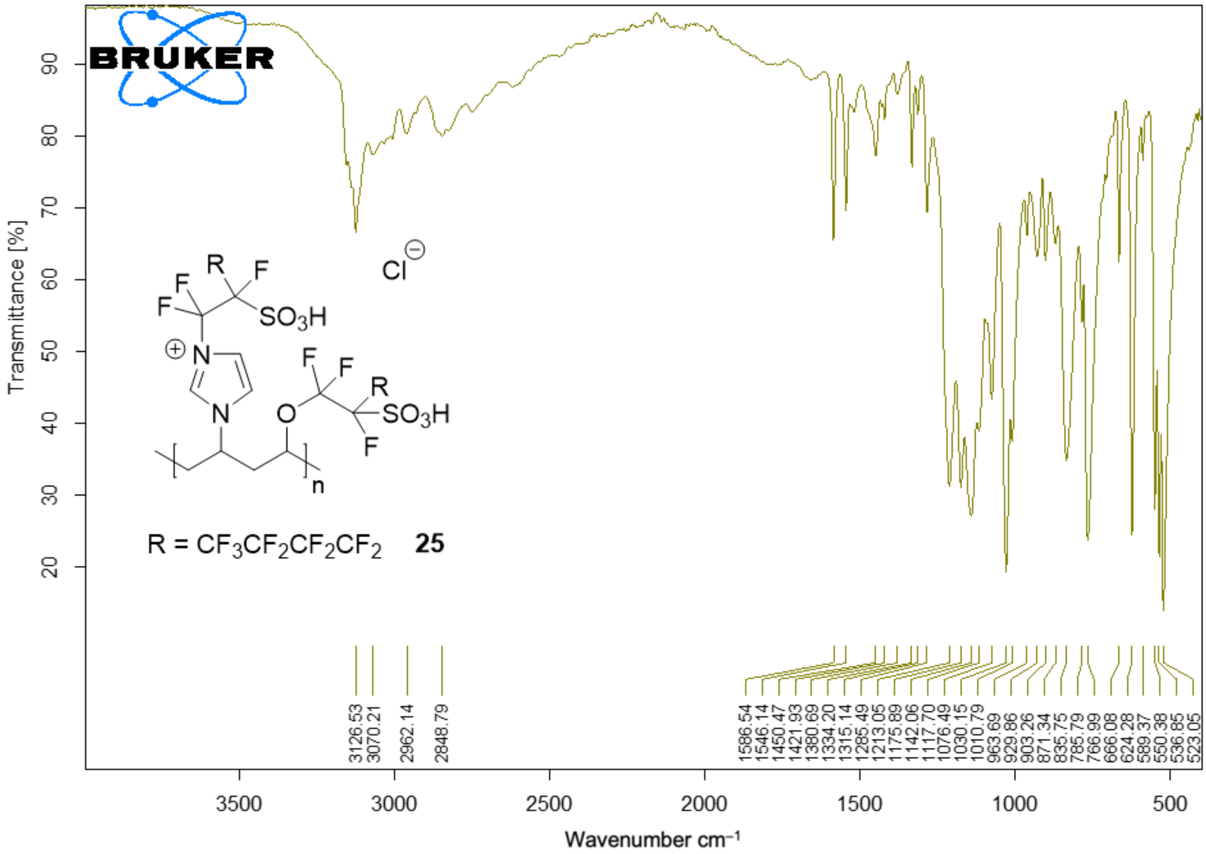

Figure S19. IR spectrum of copolymer V (Co-NVIm-PVA-12F) (**25**)
